# A Family of Human MicroRNA Genes from Miniature Inverted-Repeat Transposable Elements

**DOI:** 10.1371/journal.pone.0000203

**Published:** 2007-02-14

**Authors:** Jittima Piriyapongsa, I. King Jordan

**Affiliations:** School of Biology, Georgia Institute of Technology, Atlanta, Georgia, United States of America; Temasek Life Sciences Laboratory, Singapore

## Abstract

While hundreds of novel microRNA (miRNA) genes have been discovered in the last few years alone, the origin and evolution of these non-coding regulatory sequences remain largely obscure. In this report, we demonstrate that members of a recently discovered family of human miRNA genes, hsa-mir-548, are derived from Made1 transposable elements. Made1 elements are short miniature inverted-repeat transposable elements (MITEs), which consist of two 37 base pair (bp) terminal inverted repeats that flank 6 bp of internal sequence. Thus, Made1 elements are nearly perfect palindromes, and when expressed as RNA they form highly stable hairpin loops. Apparently, these Made1-related structures are recognized by the RNA interference enzymatic machinery and processed to form 22 bp mature miRNA sequences. Consistent with their origin from MITEs, hsa-mir-548 genes are primate-specific and have many potential paralogs in the human genome. There are more than 3,500 putative hsa-mir-548 target genes; analysis of their expression profiles and functional affinities suggests cancer-related regulatory roles for hsa-mir-548. Taken together, the characteristics of Made1 elements, and MITEs in general, point to a specific mechanism for the generation of numerous small regulatory RNAs and target sites throughout the genome. The evolutionary lineage-specific nature of MITEs could also provide for the generation of novel regulatory phenotypes related to species diversification. Finally, we propose that MITEs may represent an evolutionary link between siRNAs and miRNAs.

## Introduction

Numerous human genome transcripts lack protein coding capacity, and these non-coding RNA (ncRNAs) perform a variety of structural, enzymatic and regulatory functions [1]. MicroRNAs (miRNAs) are a class of short ∼22nt ncRNA that function as post-transcriptional regulators of gene expression [2]. Mature miRNAs are processed from longer RNA sequences that form local stem-loop (hairpin) structures [3]. The first step of the miRNA biogenesis pathway occurs in the nucleus where the RNase III enzyme Drosha cleaves both strands of the so-called pri-miRNA at the base of the stem. This yields a ∼70–90 bp pre-miRNA hairpin that is exported to the cytoplasm where it is further processed by Dicer, another RNase III endonuclease. Dicer recognizes the double stranded portion of the RNA close the base of the pre-miRNA stem and cleaves both strands of the duplex in two places. This reaction cuts off the loop portion of the molecule as well as the terminal part of the stem leaving a short duplex that consists of the mature miRNA and a complementary miRNA* sequence that is rapidly degraded. Once liberated in this way, the mature miRNA sequence binds to partially complementary target sites in the 3′ untranslated regions (UTRs) of messenger RNAs (mRNAs) and regulates expression through a process of mRNA degradation and/or translational repression [3].

miRNAs were only recently discovered [4], and details regarding their origin and evolution have yet to be fully worked out. Since their original discovery, miRNAs have been detected in all metazoa surveyed for their presence [3]. However, the full-extent of miRNA genes in any particular genome is unknown, and a number of studies aimed at the detection of novel miRNA genes have been conducted to address this issue. Bioinformatic miRNA discovery relies primarily on the sequence conservation of miRNA genes and secondary structure of the pre-miRNAs [5], while experimental efforts consist of forward [4] and reverse [6] genetic studies as well as efforts to clone short mature miRNA sequences [7]–[9]. Cloning mature miRNA sequences is technically challenging given their small size and associated instability. Thus, direct miRNA cloning is not well suited to large scale discovery efforts and may have already reached the point of diminishing returns [7]. A recently published report described a novel high-throughput miRNA cloning technique aimed at increasing the efficiency of miRNA discovery [10]. This technique is based on the serial analysis of gene expression (SAGE) and takes advantage of well established protocols tailored to small RNA sequences. Application of this SAGE-based approach to human transcripts confirmed the presence of numerous miRNA genes that had been detected previously through computational and/or experimental surveys and also yielded more than 100 novel miRNA sequences [10]. Including these new data, version 8.2 of miRBase, the online microRNA database [11], reports 462 human miRNA genes. The importance of miRNAs for human gene regulation is underscored by target site predictions [12], which reveal that these human miRNAs have the potential to regulate thousands of human genes.

miRNAs are closely related to another class of ncRNA, known as small interfering RNAs (siRNA), in terms of both biogenesis and regulatory function [3], [13]. The mature biologically active forms of siRNA and miRNA are both processed from double stranded RNA (dsRNA) by Dicer. However, siRNAs are generated from long dsRNA precursors, which can be either endogenous or exogenous transcripts, whereas mature miRNAs are processed from shorter endogenous transcripts that form local hairpin structures. Numerous siRNA molecules are processed from both strands of the long dsRNA precursor, whereas a single mature miRNA sequence is generated from only one strand of the pre-miRNA hairpin. While miRNAs can act through translational repression of their targets, they may also cause mRNA degradation of their target genes in the same way that siRNAs do [14]–[17].

One previously recognized distinction between these two classes of regulatory RNA is the fact that miRNAs are generally found in unique genomic loci, such as intergenic regions [3], while siRNAs originate from within already characterized sequences such as genes and transposable elements (TEs) [18]–[20]. However, a recent report indicated that a number of mammalian miRNAs, including six human miRNAs, are in fact derived from TEs [21]. The abundance and repetitive nature of TE sequences could provide a natural mechanism for the generation of multiple miRNA genes, along with homologous target sites, dispersed throughout the human genome. TEs may also provide an evolutionary connection between siRNAs and miRNAs. In light of these possibilities, we sought to investigate the relationship between human miRNAs and TEs by evaluating whether there exist families of related (paralogous) miRNA genes that are derived from TE sequences. We compared the genomic locations of experimentally characterized human miRNA genes to the annotated human TE sequences and discovered a set of closely related miRNA genes derived from a family of miniature inverted repeat transposable elements (MITEs). The palindromic sequence structure of MITEs, considered together with their insertion into transcriptionally active regions of the human genome, suggests a specific mechanism by which these kinds of elements could give rise to emergent mature miRNAs.

## Methods

### TE-miRNA sequence analysis

The UCSC Genome and Table Browsers [22], [23] were used to analyze the March 2006 human genome reference sequence (http://www.genome.ucsc.edu/cgi-bin/hgGateway?org = Human&db = hg18). This sequence is referred to as the hg18 assembly on the UCSC Genome Bioinformatics website and corresponds to the human genome build 36.1 assembled by the National Center for Biotechnology Information (NCBI). The Table Browser was used to search genome-wide for co-located TE and miRNA gene sequences, and the Genome Browser was used to visualize the results on a case-by-case basis. The genome locations and identities of human TE sequences were taken from annotation generated by the RepeatMasker program (http://www.repeatmasker.org) [24]. The genome locations and identities of experimentally characterized human miRNA gene sequences were taken from release 8.2 of the miRBase sequence database (http://microrna.sanger.ac.uk/sequences/) [11]. Evolutionary conservation between human Made1-derived miRNA gene sequences and six mammalian genomes – chimp, rhesus, mouse, rat, dog and cow – was assessed based on the Alignment Net track of the UCSC Genome browser, which shows the best pairwise between-genome alignments corresponding to orthologous regions [25].

The sequences of Made1-derived miRNAs were compared to the human genome sequence using the BLAT program [26]. Homologous genomic sequences were counted as statistically significant hits that matched ≥80% of the length of the query miRNA sequence and were confined to a local genomic region no longer than 120% of the query length (*i.e.* long genomic insertions were not counted). Made1 and hsa-mir-548 sequences were aligned to each other using the program ClustalW [27]. NCBI's BLASTN program (http://www.ncbi.nlm.nih.gov/BLAST/) [28] was used to search the Expressed Sequence Tags Database (dbEST) [29] for expressed human MITE sequences. Human genomic expression data from Affymetrix tiling GeneChips [30], represented in the UCSC Genome Browser, were evaluated in order to identify transcriptionally active regions of the human genome. RNA sequences were folded using the Mfold [31] web server (http://www.bioinfo.rpi.edu/applications/mfold/rna/form1.cgi).

### Regulatory analysis

Putative miRNA target sites were taken from the miRBase Targets website (http://microrna.sanger.ac.uk/targets/v3/), which uses a modified implementation of the miRanda algorithm [12]. 3′ UTRs of Ensembl genes were also searched for Made1 derived target sites. In this case, the same approach used by the current miRBase implementation of miRanda for annotating 3′ UTRs was employed. Specifically, if there is no hexamer of ‘A’ residues in the last 30 bp of the 3′ UTR, the sequence is extended 2,000 bp. The random expectation for the number of target genes identified by both methods was calculated by taking their joint probability multiplied by the total number of human genes (n = 23,269 from Ensembl version 41). The joint probability was calculated by multiplying the relative human genome frequencies of each target set. The difference between the expected and observed number of target genes identified using both methods was calculated using the binomial distribution.

Comparative genomic sequence data from the UCSC genome browser were used to analyze the relative evolutionary conservation levels for predicted hsa-mir-548 target sites. Position-specific conservation scores were derived from multiple whole genome sequence alignments between the human and 16 other vertebrate genomes [25], [32]. The scores correspond to the posterior probability that a human genome site is conserved as determined by the phastCons program [33], and position-specific scores were averaged across target sites.

Human gene expression patterns across 79 tissues were taken from the Novartis Research Foundation's Symatlas [34]. Relative expression profiles for genes with hsa-mir-548 target sites were computed for each gene by dividing the gene's tissue-specific expression (signal intensity) values by the gene's median expression value over all 79 tissues and then log_2_ normalizing the resulting ratios. The program Genesis [35] was used to visualize the relative expression profiles, to group related expression profiles with k-means clustering and to group tissues with hierarchical clustering.

Clusters of coexpressed genes were analyzed with the program GOTree Machine (GOTM) [36] to look for over-represented Gene Ontology [37] functional annotations. To do this, genes in each cluster were annotated with their biological process GO terms. The frequencies of these terms were then compared to their expected frequencies based on their occurrences in the human genome, and statistically over-represented terms were identified using the hypergeometric test. Statistically over-represented terms were then mapped to the GO directed acyclic graph.

## Results and Discussion

### A TE-derived miRNA gene family

When we compared the genomic locations of experimentally characterized human miRNA sequences stored in miRBase [11] to the locations of human TEs characterized by the program RepeatMasker [24], we found that seven closely related miRNA genes (hsa-mir-548) were co-located with dispersed members of a single family of TEs known as Made1 (Table 1). These hsa-mir-548 miRNA genes were recently characterized by mapping mature cloned miRNA sequences to the human genome sequence [10]. The hsa-mir-548 mature miRNAs meet both the expression and biogenesis criteria that were articulated to ensure the accurate identification of miRNAs and the distinction between miRNAs and siRNAs [13]. In particular, the mature hsa-mir-548 miRNAs are all 22nt in length, they were identified from a cDNA library made of size fractionated RNA and they map precisely to genomic regions that are predicted to form local hairpin structures.

**Table 1:** Made1 derived miRNA genes in the human genome

Name^1^	Accn^2^	Chr^3^	Start^3^	Stop^3^	Str^3^	Duplicates^4^
hsa-mir-548a-1	MI0003593	6	18679994	18680090	+	24
hsa-mir-548a-2	MI0003598	6	135601991	135602087	+	81
hsa-mir-548a-3	MI0003612	8	105565773	105565869	−	82
hsa-mir-548b	MI0003596	6	119431911	119432007	−	23
hsa-mir-548c	MI0003630	12	63302556	63302652	+	124
hsa-mir-548d-1	MI0003668	8	124429455	124429551	−	71
hsa-mir-548d-2	MI0003671	17	62898067	62898163	−	145

1 miRNA gene name

2 miRBase accession number

3 Human genome chromosome coordinates and strand information

4 Duplicate sequences taken as the number of statistically significant human genome BLAT hits that also pass the match length criteria described in the Methods section

**Figure 1:**
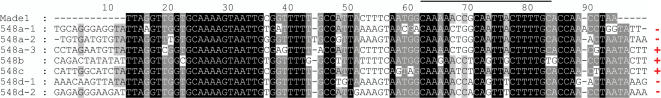
Multiple sequence alignment of Made1 and hsa-mir-548 genes. The location of the mature miRNA sequence is indicated by the bar over the alignment. The strand of the Made1 element (+/−) from which the miRNA genes are derived is shown to the right of the alignment.

Inspection of the multiple sequence alignment of a full length Made1 sequence with all seven hsa-mir-548 miRNAs provides clear evidence that the miRNAs are in fact derived from the Made1 elements (Figure 1). Individual hsa-mir-548 sequences were queried against the human genome sequence to search for duplicates. Each hsa-mir-548 gene showed significant similarity to numerous genomic regions (Table 1), suggesting the possibility that this miRNA gene family may include many as yet uncharacterized members.

**Figure 2:**
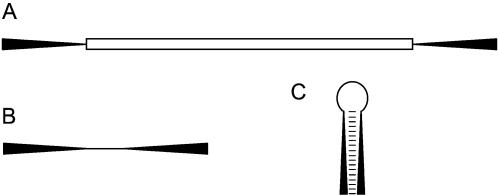
Schematic illustrating the relationship between Hsmar DNA-type TEs (A), Made1 MITEs (B) and hairpins (C) of the kind recognized by the miRNA enzymatic processing machinery. A) A full length DNA-type element with terminal inverted repeats (TIRs) flanking an open reading frame (ORF) is shown. B) Non-autonomous MITE derivative of a full length DNA-type element, containing TIRs but no internal ORF. C) Predicted hairpin structure that would be formed by base-pair interactions of the MITE TIRs.

Made1 elements were independently characterized by several groups as non-autonomous derivatives of the human mariner-like transposable element (TE) Hsmar1 [38]–[40]. Hsmar1 elements are DNA-type TEs, approximately 1,300 bp in length, which possess a transposase-encoding open reading frame flanked by terminal inverted repeat (TIR) sequences (Figure 2A) [41]. Related Made1 elements are only 80bp long with two 37 bp TIRs and a 6 bp intervening region (Figure 2B). In this sense, Made1 sequences are palindromes, and if they were to be transcribed, they would form highly stable hairpin-loops reminiscent of the pre-miRNA structures that are processed to form mature miRNAs (Figure 2C).

The formation of TIR-based dsRNA hairpins from Made1 would require the generation of full-length (or nearly so) element transcripts. The human expressed sequence tag (EST) database was searched using BLASTN [28], with a full-length Made1 query sequence, to test for this. We found 141 human ESTs that showed >80% sequence similarity to the Made1 query sequence over >80% of the length of the element (Table S1). Furthermore, the EST analysis indicates that Made1 sequences are widely expressed in a variety of tissue-types, providing ample opportunity for the formation of mature miRNAs.

Interestingly, Made1 transcripts destined to become hsa-mir-548 miRNAs are generated from both strands of the element (Figure 1). Because the element sequences are palindromes, transcripts produced in either orientation (+/−) would yield local hairpin structures. Indeed, the only difference between strand-specific transcripts is seen for the intervening 6bp sequence that forms the loop in the structure (positions 51–56 in Figure 1). This suggests that Made1 expression may result from read-through transcripts promoted from adjacent genomic positions, as opposed to a strand specific promoter encoded by the element itself. Consistent with this notion, we found that a number of Made1 homologous ESTs include substantial upstream and downstream sequences (Table S1).

**Figure 3:**
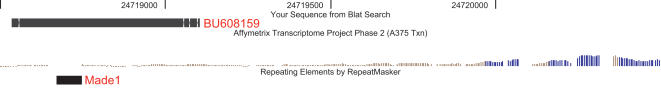
Made1 insertion in a transcriptionally active region of the human genome. The Made1 element shown is expressed by read-through from an adjacent promoter position in the genome. The EST BU608159 consists of the Made1 element along with 678 bp of flanking DNA.

**Figure 4:**
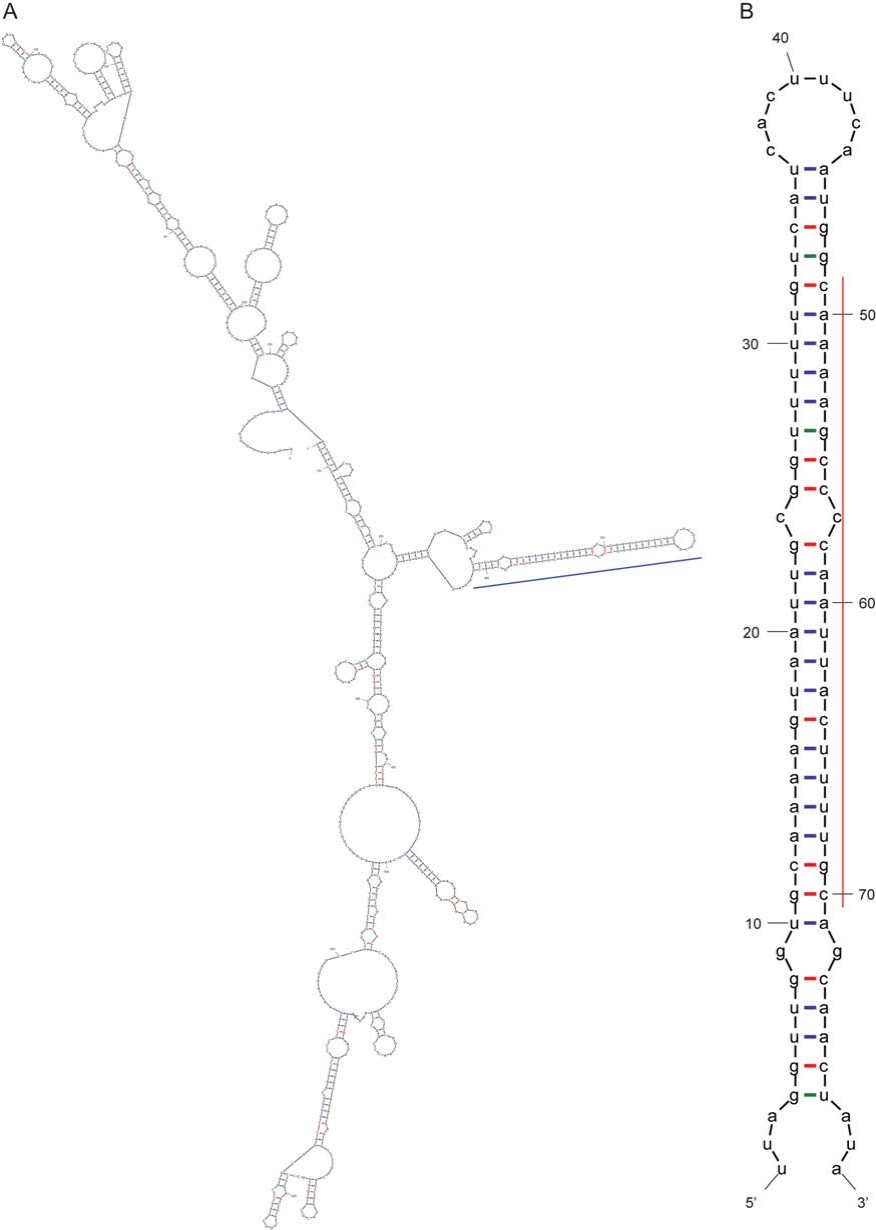
RNA secondary structures of the entire BU608159 EST (A) and the Made1 element contained within this transcript (B). The Made1 hairpin region of the BU608159 structure is indicated with a blue bar (A), and the location of the mature miRNA sequence is shown with the red bar (B).

Therefore, we propose a model whereby Made1 insertions into transcriptionally active genomic regions would yield viable pri-miRNA structures that would be processed into mature miRNA sequences by the RNA interference enzymatic machinery. An example of such a scenario can be seen for the human EST corresponding to the Genbank accession BU608159. This 754 base pair (bp) EST maps to chromosome chr13 at positions 24,718,360–24,719,104; it includes a nearly full length Made1 element as well as 325 bp of 5′ flanking DNA and 353 bp of sequence 3′ to the element. Visualization of genomic expression data, generated with human genome tilling arrays [30], shows that this particular Made1 is inserted into an intergenic region of the genome that is transcriptionally active (Figure 3). In this case, the entire Made1 element is transcribed as a read-through initiated from an adjacent genomic position. When the RNA structure of the EST, which includes the Made1 element along with expressed genomic flanking regions, is evaluated using the program Mfold, the Made1 region can be seen to form the most stable stem-loop structural element in the RNA (Figure 4A). The tight hairpin formed by the element is similar to the structures processed by Drosha and Dicer, and the location of the mature miRNA sequence, in the stem close to the 3′ end of the structure, is consistent with the mode of cleavage thought to be employed by the Dicer (Figure 4B).

### Regulatory effects of hsa-mir-548

Mature miRNA sequences associate with the RNA-induced silencing complex (RISC), which facilitates their regulatory interactions with target mRNAs (mRNAs) [3]. miRNAs wield specific regulatory effects on gene expression through physical interactions with partially complementary sequences in the 3′ untranslated regions (UTRs) of their target genes' transcripts. We sought to characterize the potential regulatory and functional effects of hsa-mir-548 miRNAs by analyzing the genes that they are predicted to target.

Putative hsa-mir-548 target sites were identified using two methods: i-by the modified miRanda algorithm implemented in miRBase and ii-by searching 3′ UTRs for Made1 sequences that are complementary to the mature hsa-mir-548 miRNAs. According to the miRBase predictions, the seven hsa-mir-548 genes have 3,527 potential target genes. Made1 related targets, on the other hand, are found in only 179 genes. This was slightly surprising given that there are 7,850 annotated Made1 sequences in the human genome. When the search for Made1 derived target sites was extended to entire transcripts, only one additional target was found in a 5′ UTR. Apparently, Made1 sequences avoid protein coding gene exon regions and thus are poorly represented among potential hsa-mir-548 target sites. Furthermore, the intersection of the target gene sets derived from the miRBase versus Made1 consists of a mere 29 genes, and this figure is only slightly higher than the random expectation of 27 shared targets (*P* = 0.07 binomial distribution). That both target prediction methods detect such a small number of the same targets can be attributed to the fact that Made1 targets are likely to be avoided by the miRanda based approach due to its criterion of sequence conservation and the fact that Made1 is an evolutionarily young TE family. Indeed, when the sequence conservation levels of target sites identified by the two methods were compared, Made1 related targets were found to be significantly less conserved, on average, than miRanda predicted targets (conservation scores: Made1 targets = 0.082±0.017 miRanda targets = 0.319±0.007; t = 11.27 *P* = 5.7e-29 Student's ttest).

**Figure 5:**
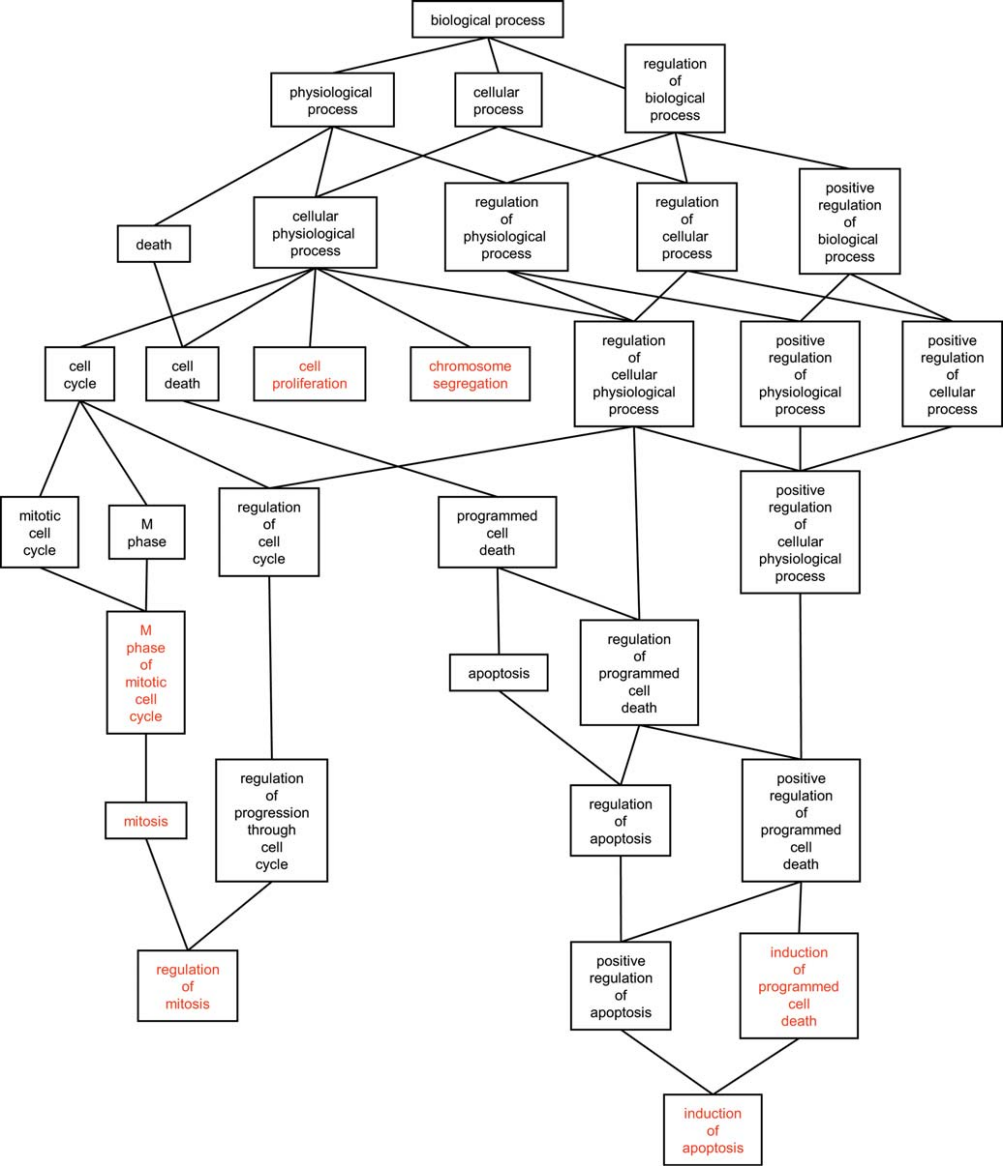
GO biological process terms over-represented among the set of genes with Made1 derived hsa-mir-548 target sites. The portion of the directed acyclic graph (DAG) containing all paths from the root biological process term to the over-represented functional category terms is shown. Over-represented functional categories are indicated in red.

The potential functional relevance of genes with Made1 derived target sites was evaluated by considering their Gene Ontology (GO) biological process annotations and looking for over-represented functional categories. This procedure identified seven over-represented GO biological process categories that include a total of 11 genes (Table S2). The relationships among the over-represented GO functional categories in the Made1 target gene set can be visualized on the GO directed acyclic graph (Figure 5). This set includes genes with functional roles in cell proliferation, mitosis and apoptosis, all categories that are related to cancer. The hsa-mir-548 genes were characterized by virtue of their expression in colorectal cancer cell lines and tissue samples [10]. If hsa-mir-548 expression is upregulated in colorectal cancer tissue, it may lead to the repression of genes that normally control cellular proliferation. Consistent with this scenario, several of the genes that correspond to over-represented functional categories were found to be down regulated in colorectal cancer tissue (Table S2). These include genes encoding a cell division cycle protein (ENSG00000004897), a C epsilon type protein kinase (ENSG00000171132) and a centromere/kinetochore protein (ENSG00000086827).

As mentioned previously, the paucity of Made1 related target sites was somewhat unexpected. Nevertheless, the identification of numerous non-Made1 related target sites is interesting in the sense that it suggests that TE-derived miRNAs may be able to regulate host genes that do not have any related TE sequences. There are two models to explain the repressive effects that miRNAs exert on target gene expression: i-translational repression and ii-mRNA degradation [16], [17], [42]. Recently, anti-correlated expression patterns between miRNA sequences and their target mRNAs have provided evidence in favor of the mRNA degradation model [43]. We sought to further evaluate the potential mRNA degradation-based regulatory effects of the hsa-mir-548 miRNAs by searching for down regulation of putative target genes in tissue samples similar to the colorectal samples from which they were cloned [10]. Consideration of target gene relative expression levels can also be used to help validate target site predictions, which are prone to false positives.

**Figure 6:**
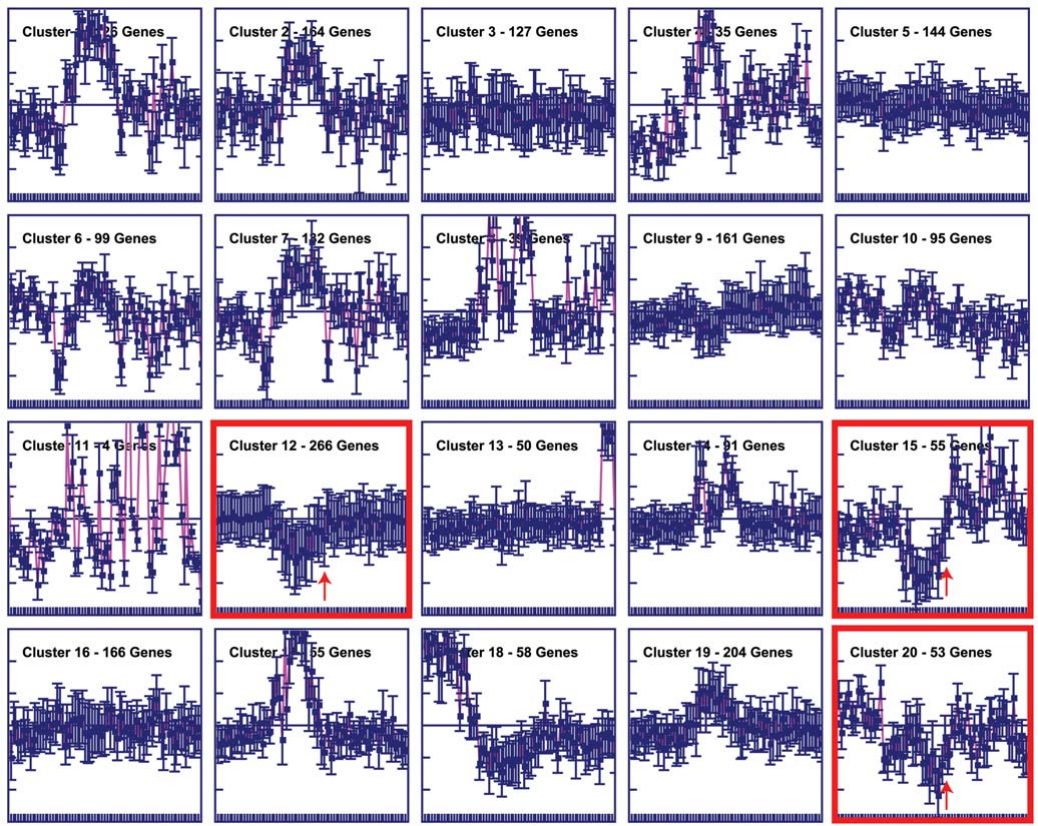
Coexpressed clusters of putative hsa-mir-548 target genes. Centroid views with average tissue-specific expression values are shown for all 20 clusters. Clusters containing genes down-regulated in the colorectal adenocarcinoma sample are shown in red and arrows indicate the colorectal sample.

**Figure 7:**
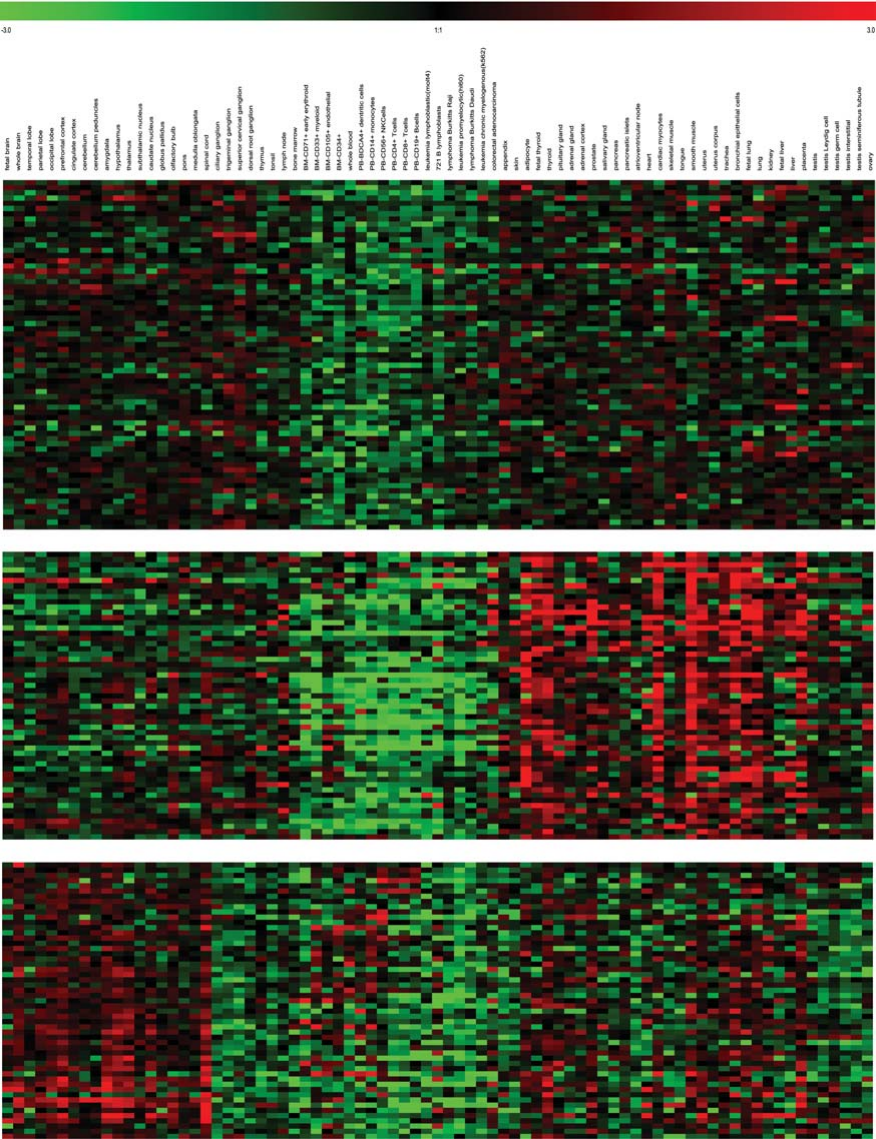
Representative gene expression profiles for putative hsa-mir-548 target genes from three coexpressed clusters (12, 15 and 20 in Figure 6). Expression profiles are median centered and log_2_ normalized, and the log_2_ ratio color scale is shown above the plot. Overexpressed genes are shown in red and underexpressed genes are shown in green.

**Figure 8:**
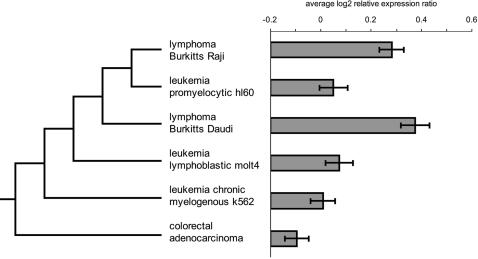
Relationships and average relative expression levels among the cancer tissues samples from the Novartis Symatlas microarray dataset.A dendogram relating the cancer samples based on similarities (differences) among relative expression levels is shown along with the average relative expression levels for all genes with hsa-mir-548 target sites in each of the cancer samples.

Gene expression profiles for potential hsa-mir-548 targets were taken from the Novartis Research Foundation's Symatlas [34]. For the miRBase set, a total of 2,045 target genes were found with corresponding Symatlas expression data across 79 human tissues. The expression data were median and log normalized to yield relative tissue-specific gene expression profiles, and these profiles were separated into 20 co-expressed groups of genes using k-means clustering. Three of these clusters – 12, 15 and 20 – showed marked down-regulation of the colorectal adenocarcinoma sample (Figure 6). Interestingly, the genes found in these same clusters tended to be down-regulated in all five of the other cancer-related samples in the data set (Figure 7). This suggests the possibility that hsa-mir-548 miRNA genes may play some global role related to the regulation of gene expression in cancer. Indeed, hierarchical clustering of the tissue-samples based on the gene expression data unites all of the cancer samples into a single group to the exclusion of all normal tissues (Figure S1); however, the colorectal adenocarcinoma sample is the outlier of this group (Figure 8). When the log_2_ median expression ratios were averaged for all genes with putative hsa-mir-548 target sites, the colorectal sample had the lowest relative expression level (*q* = 9.72, *v* = 12738, *k* = 6, *P*<0.001 Tukey test; Figure 8). This finding is consistent with the fact that the hsa-mir-548 genes were isolated from colorectal cancer samples, and points to an additional more specific role for these genes in colorectal cancer related gene regulation. The functional affinities of the genes in the three down regulated clusters were assessed using the same GO-based approach as for the set of genes with Made1 target sites. There are 29 GO biological process categories, encompassing 104 genes, which contain an over-representation of genes from these clusters (Table S3). These include genes involved in cell adhesion, cell signalling and signal transduction. The positions of these categories on the GO biological process DAG can be seen in Figure S2.

We also compared putative hsa-mir-548 target genes to a recently published collection of genes that were indicated as being involved in colorectal cancer by microarray expression profiling [44]. We found 22 examples of putative hsa-mir-548 target genes that were previously found to be related to colorectal cancer based on down-regulation in six separate microarray studies (Table S4). These include a number of genes encoding various immune cell receptors as well as transcription factors and tumor necrosis factors. The apparent connection between cancer and the immune system in our dataset is supported by the similar down-regulated expression patterns seen for hsa-mir-548 target genes among the cancer and immune tissue samples (Figure 7). However, a number of genes previously implicated in colorectal cancer etiology by virtue of up-regulation in previous studies were also found to have predicted hsa-mir-548 target sites. These cases may represent false positive target site predictions or could point to instances where hsa-mir-548 miRNAs act through translational repression and thus do not repress mRNA expression levels.

### Conclusion

We report here a human miRNA gene family derived from TEs. The palindromic structure of the Made1 elements from which the hsa-mir-548 miRNA genes originated, together with their insertion into transcriptionally active genomic regions, points to a specific mechanism by which these sequences can be recognized and processed by the enzymatic machinery that yields mature miRNA sequences. In addition, the dispersed repetitive nature of TE sequences provides for the emergence of multiple novel miRNA genes as well as numerous homologous target sites throughout the genome.

TEs also tend to be among the most lineage-specific, *i.e.* recently evolved, sequences in the human genome [45]. Made1 elements emerged along the primate evolutionary lineage, and orthologous hsa-mir-548 sequences are confined to the human, chimpanzee and rhesus macaque genome sequences (Figure S3). While many miRNA genes are conserved across more distantly related species, a recent analysis of the human genome detected numerous putative miRNAs that are not evolutionarily conserved [5]. TEs, such as Madel, represent a natural source of such lineage-specific miRNAs, which could in turn be responsible for regulatory phenotypes that contribute to evolutionary diversification between species. The relatively low conservation of Made1 derived target sites is also consistent with this lineage-specific mode of evolution.

MITEs are widely distributed among eukaryotes [46] and could provide for the emergence of regulatory RNAs, such as miRNAs, siRNAs or other small non-coding RNAs, in many different genomic contexts. For instance, MITEs are particularly prevalent in plants where they were first discovered [47]; the rice genome alone contains ∼90,000 MITEs [48]. A striking feature of plant MITEs is their apparent preference for insertion in gene regions [49], [50]. Accordingly, many thousands of plant MITEs must be expressed along with the gene sequences in which they are inserted. This would provide ample opportunities for the processing of MITE hairpins by RNA interference enzymatic machinery, which is known to play a particularly important role in plant gene regulation [51].

Finally, we would like to propose that MITEs, such as Made1, may represent an evolutionary intermediate between siRNAs and miRNAs. A number of epigenetic gene silencing mechanisms, such as cytosine methylation [52], genomic imprinting [53] and heterochromatin [54] are thought to have evolved as defense mechanisms against transposition. Subsequently, these TE silencing mechanisms were co-opted as global regulators to control the expression patterns of host genes. This may have led to the increase in regulatory and phenotypic complexity seen among members of the eukaryotic crown group. In a similar way, RNA interference by siRNAs is considered to have evolved to silence TEs [18], [20]. Consistent with this model, there are a number of cases of siRNAs that originate from TEs in different species [55]–[58]. Perhaps the best characterized example of this is the Muk TE repressor in maize [19]. Muk is an effective silencer of the MuDR DNA-type TE, and the Muk locus consists of an inverted duplication of a partially deleted MuDR element. When Muk is transcribed, it yields a long (>2 kb) dsRNA hairpin structure that is processed to yield siRNAs. The connection between TEs and siRNAs has led to the proposal that origination from TEs distinguishes siRNAs from miRNAs [3]. However, as reported here and elsewhere [21], more and more TE-derived miRNAs are being discovered.

The model of miRNA emergence from MITEs that we propose here (Figure 2) suggests a way that miRNAs could have evolved from TE encoded siRNAs. One possible source of the TE encoded dsRNAs that serve as siRNA precursors is snap back panhandle structures between TIRs of autonomous DNA-type elements [20]. Such panhandle structures would include long internal loop regions that correspond to the internal open reading frames that are lost when autonomous elements are converted to non-autonomous MITE derivatives. MITEs retain the TIRs, and those same TIRs that were processed from longer RNAs to form siRNA could be similarly processed to form miRNAs. The shorter hairpin structures formed by MITE transcripts could lead to steric constraints that result in the liberation of only one mature miRNA sequence as opposed to the numerous siRNAs that are produced from longer dsRNAs. In this way, short hairpin loop derived miRNAs may have evolved from TE encoded siRNAs. Many of the extant miRNA genes characterized today may have evolved beyond recognition to their progenitor TEs, while others may have originated from other genomic re-structuring mechanisms that juxtapose short inverted repeats [59].

## Supporting Information

Figure S1 Dendogram showing relationships among tissues from the Novartis Foundation Symatlas microarray dataset. Cancer tissues are indicated with the red bar.
(0.45 MB PDF)

Figure S2 Over-represented GO biological process categories among genes with miRanda predicted hsa-mir-548 target sites that map to colorectal cancer down-regulated co-expression clusters (i.e. 12, 15 & 20 in Figure 6). The portion of the directed acyclic graph (DAG) containing all paths from the root biological process term to the over-represented functional category terms is shown. Over-represented functional categories are indicated in red.
(0.06 MB PDF)

Figure S3 Made1-derived miRNA genes are primate-specific. Human genomic regions corresponding to Made1-derived miRNA genes are shown: A hsa-mir-548a-1, B hsa-mir-548-a2, C hsa-mir-548-a3, D hsa-mir-548-b, E hsa-mir-548-c, F-hsa-mir-548-d1, G-hsa-mir-548-d2. The UCSC Genome Browser is used to show the location of the Made1 elements (DNA) in the RepeatMasker track. Evolutionary comparisons between the human genome and the corresponding regions in the chimp, rhesus, mouse, rat, dog and cow genomes are shown using the species-specific Net tracks of the Genome Browser. Corresponding Made1 orthologous regions that are present in another species are indicated with a broad line, while regions that are missing in another species are shown with a thin line.
(3.44 MB PDF)

Table S1 Made1 homologous human expressed sequence tags (ESTs).
(0.32 MB DOC)

Table S2 Over-represented GO biological process categories among genes with Made1 derived hsa-mir-548 target sites.
(0.04 MB DOC)

Table S3 Over-represented GO biological process categories among genes with miRanda predicted hsa-mir-548 target sites that map to colorectal cancer down-regulated co-expression clusters (i.e. 12, 15 & 20 in Figure 6).
(0.09 MB DOC)

Table S4 Putative hsa-mir-548 target genes previously implicated as being involved in colorectal cancer by microarray expression profiling.
(0.20 MB DOC)
