# Supplementary figures and images for: A Family of Human MicroRNA Genes from Miniature Inverted-Repeat Transposable Elements
Source: PLoS One. 2007 Feb 14;2(2):e203. doi: 10.1371/journal.pone.0000203 (PMC1784062; doi:10.1371/journal.pone.0000203)

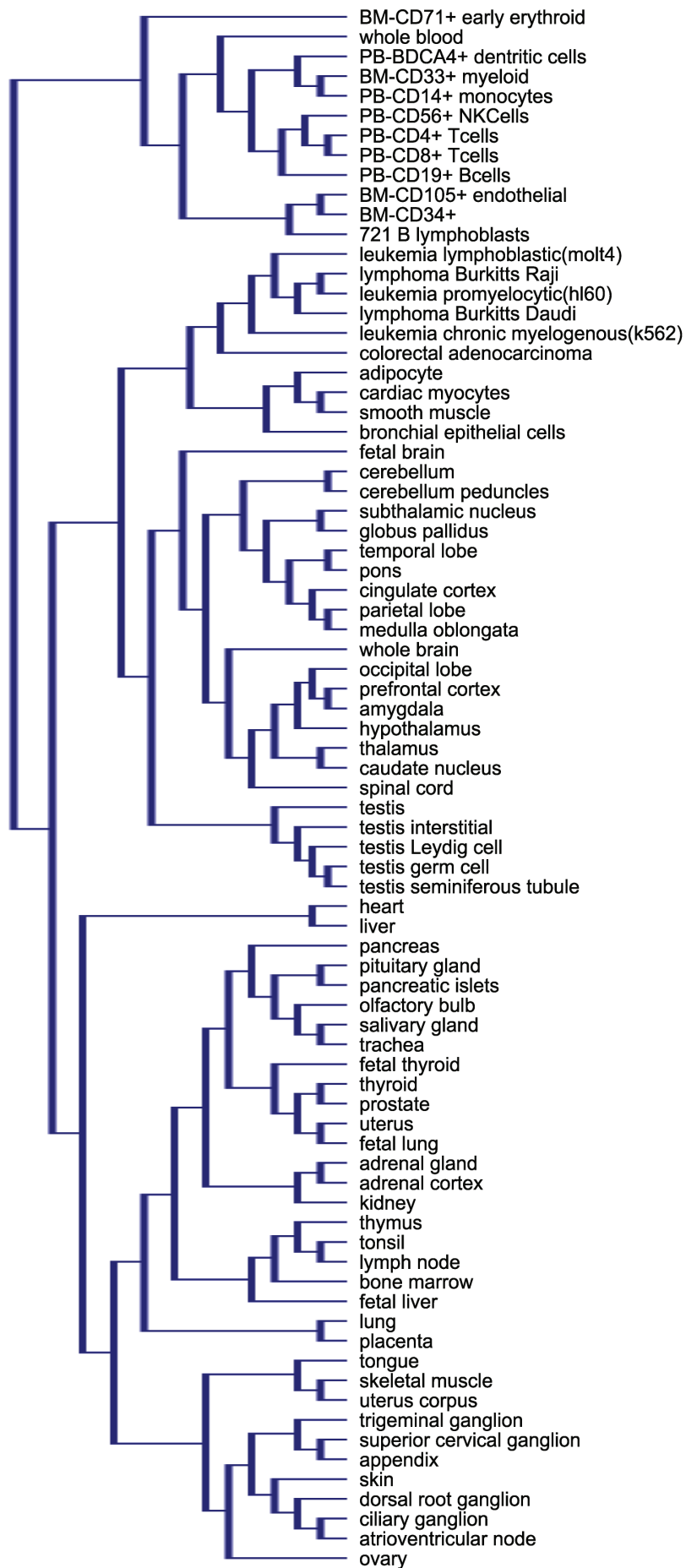

Supplement: Figure S1 — Dendogram showing relationships among tissues from the Novartis Foundation Symatlas microarray dataset. Cancer tissues are indicated with the red bar. (0.45 MB PDF) [file pone.0000203.s001.pdf]

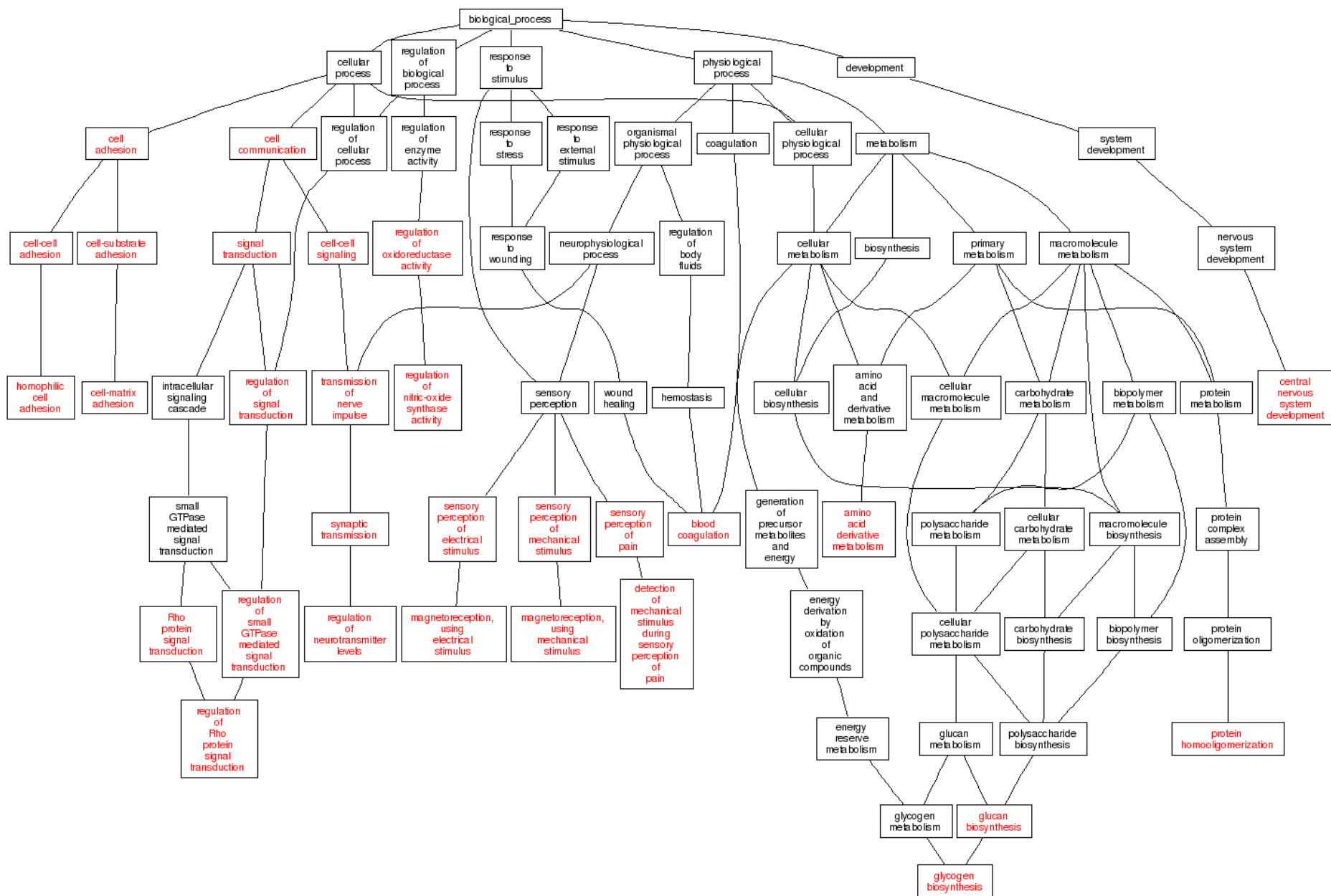

Supplement: Figure S2 — Over-represented GO biological process categories among genes with miRanda predicted hsa-mir-548 target sites that map to colorectal cancer down-regulated co-expression clusters (i.e. 12, 15 & 20 in Figure 6). The portion of the directed acyclic graph (DAG) containing all paths from the root biological process term to the over-represented functional category terms is shown. Over-represented functional categories are indicated in red. (0.06 MB PDF) [file pone.0000203.s002.pdf]

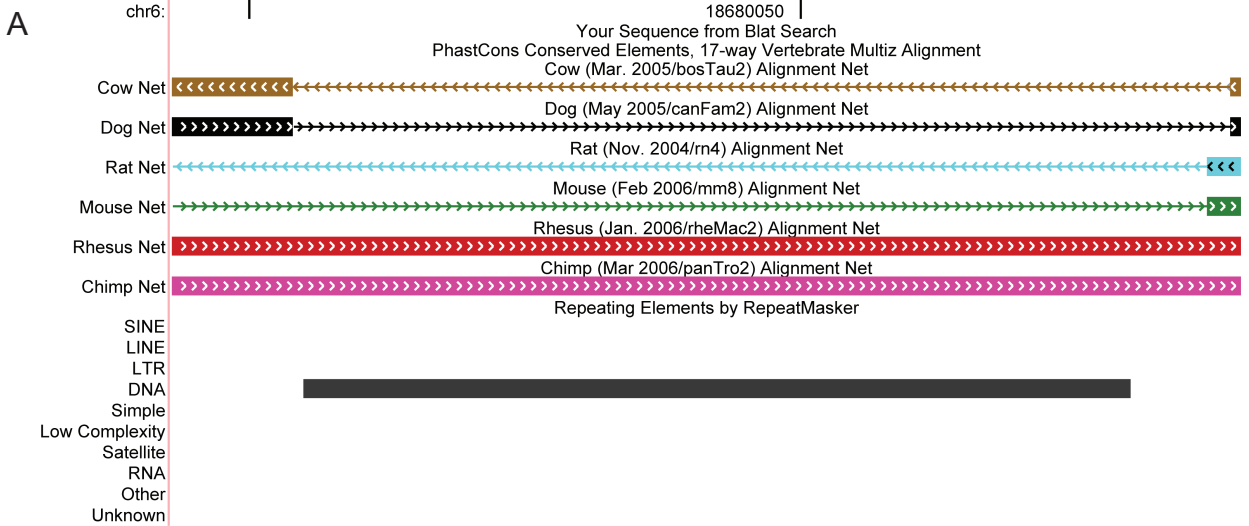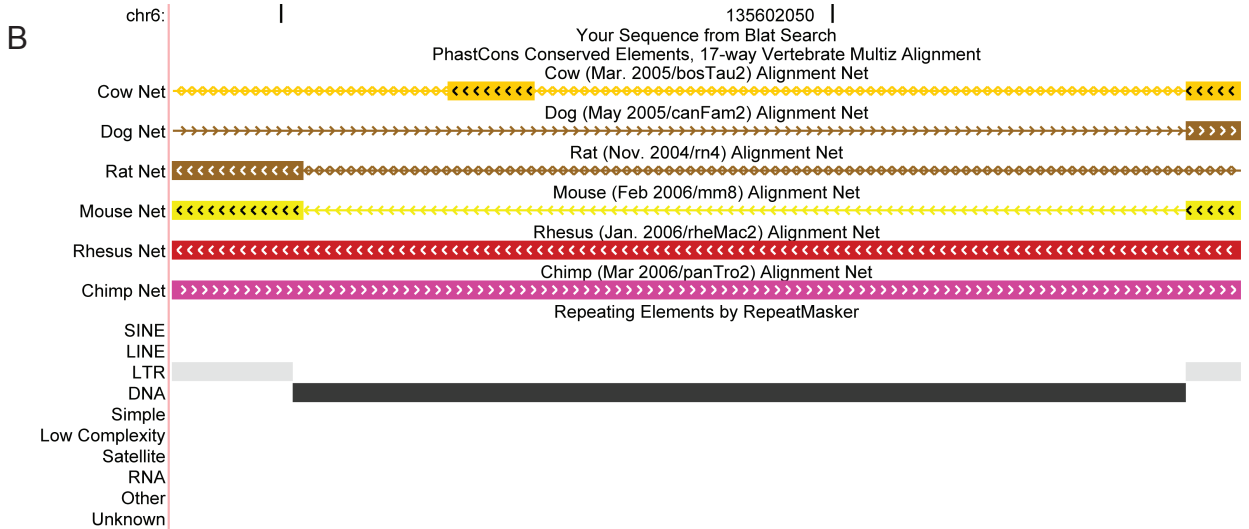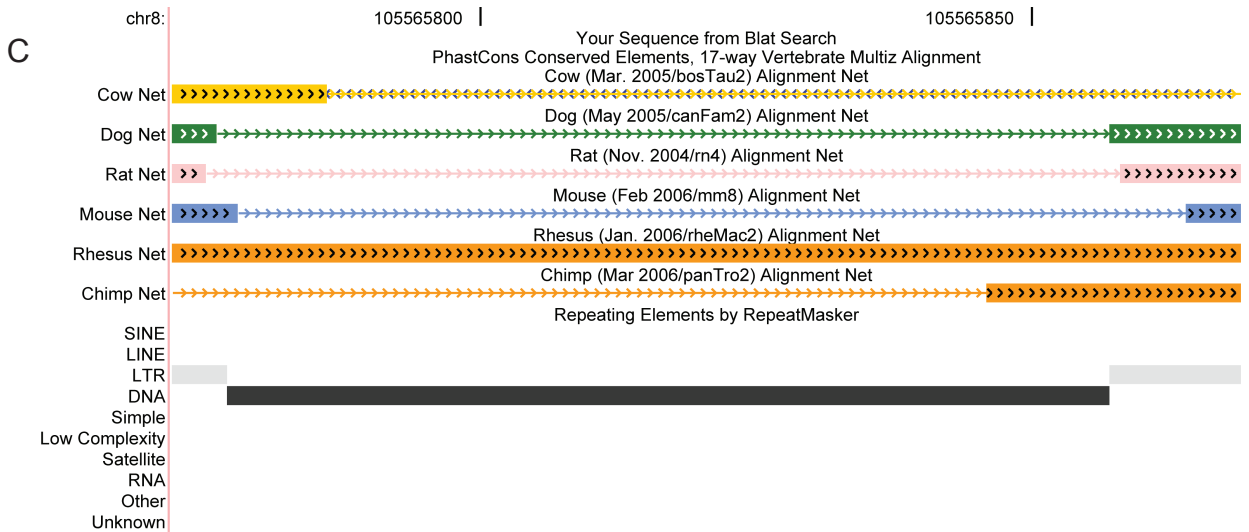

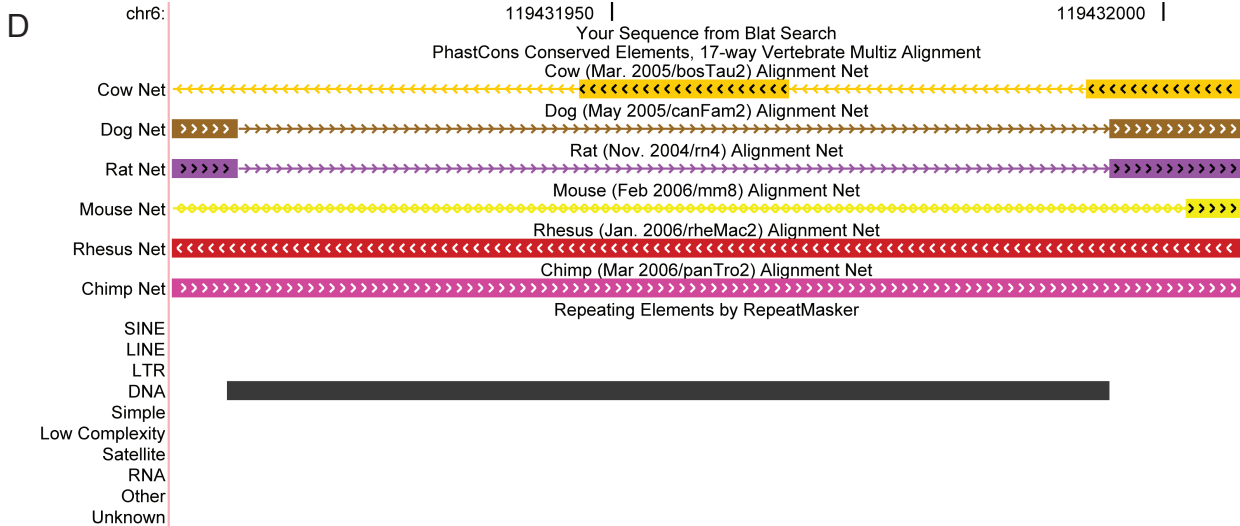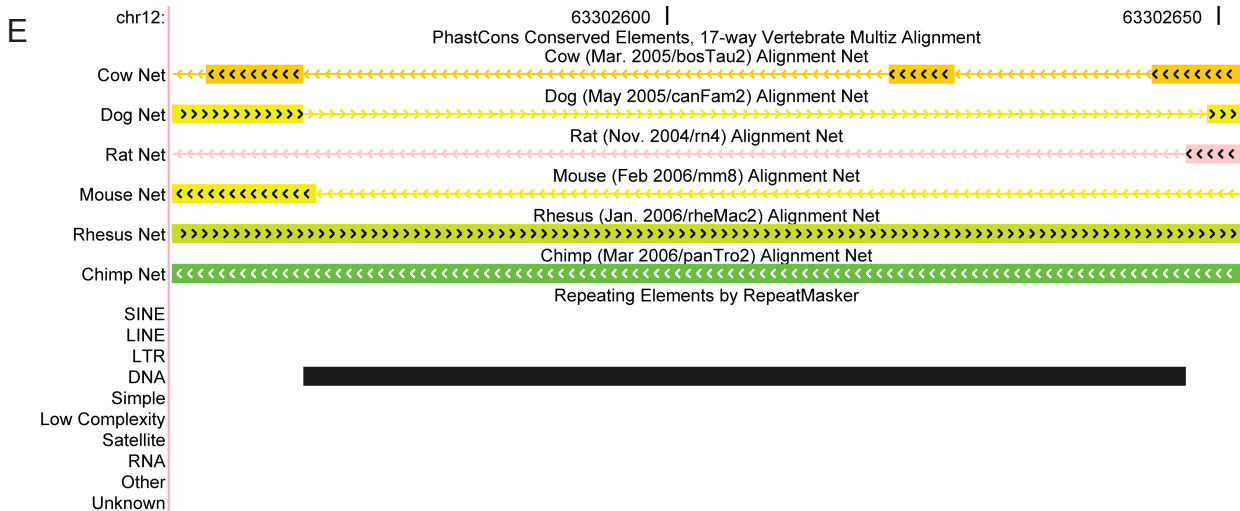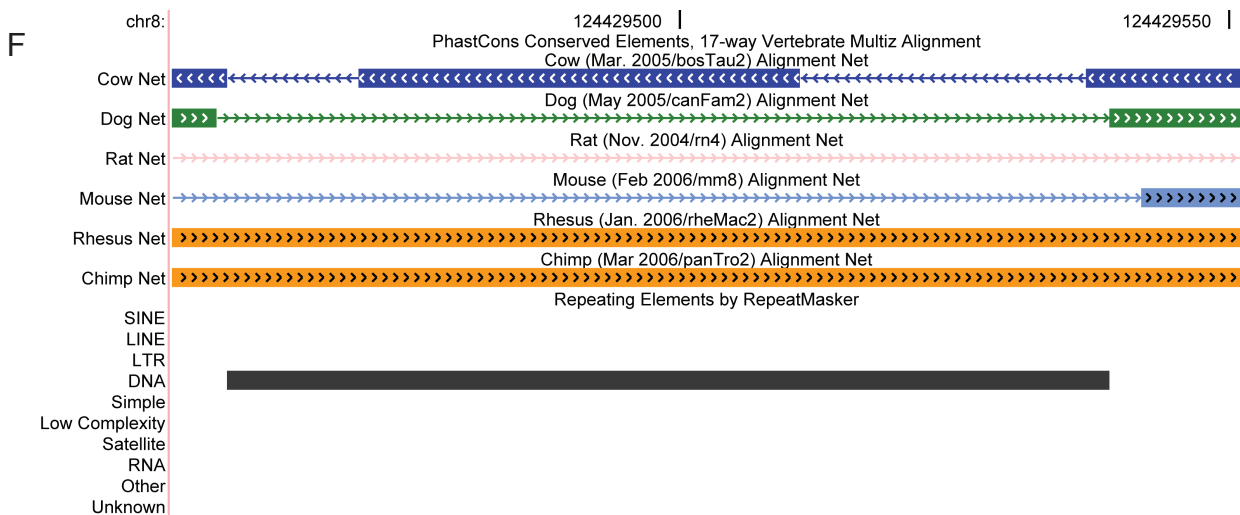

G

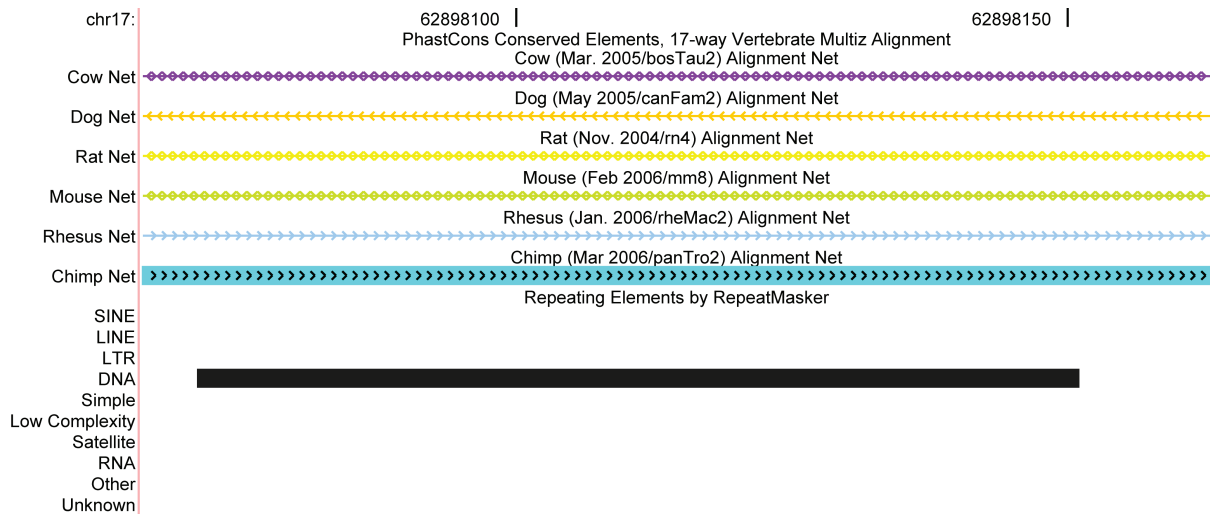

Supplement: Figure S3 — Made1-derived miRNA genes are primate-specific. Human genomic regions corresponding to Made1-derived miRNA genes are shown: A hsa-mir-548a-1, B hsa-mir-548-a2, C hsa-mir-548-a3, D hsa-mir-548-b, E hsa-mir-548-c, F-hsa-mir-548-d1, G-hsa-mir-548-d2. The UCSC Genome Browser is used to show the location of the Made1 elements (DNA) in the RepeatMasker track. Evolutionary comparisons between the human genome and the corresponding regions in the chimp, rhesus, mouse, rat, dog and cow genomes are shown using the species-specific Net tracks of the Genome Browser. Corresponding Made1 orthologous regions that are present in another species are indicated with a broad line, while regions that are missing in another species are shown with a thin line. (3.44 MB PDF) [file pone.0000203.s003.pdf]
