# Supplementary material for: A Family of Human MicroRNA Genes from Miniature Inverted-Repeat Transposable Elements
Source: PLoS One. 2007 Feb 14;2(2):e203. doi: 10.1371/journal.pone.0000203 (PMC1784062; doi:10.1371/journal.pone.0000203)
Supplement: Table S1 — Made1 homologous human expressed sequence tags (ESTs). (0.32 MB DOC) [file pone.0000203.s004.doc]

Table S1. **Made1 homologous human expressed sequence tags (ESTs)^1^.**

| **Hit identifiers** | **% identity** | **Length** | **Mis-matches** | **Gap openings** | **Query start** | **Query**  **end** | **Hit**  **start** | **Hit**  **end** | **E-value** | **Bit**  **score** |
| --- | --- | --- | --- | --- | --- | --- | --- | --- | --- | --- |
| gi|23517262|gb|BU674347.1| | 94.81 | 77 | 4 | 0 | 4 | 80 | 59 | 135 | 7.00E-26 | 121 |
| gi|11450750|gb|BF438233.1| | 93.42 | 76 | 5 | 0 | 1 | 76 | 311 | 386 | 7.00E-23 | 111 |
| gi|18976268|gb|BM668437.1| | 93.42 | 76 | 5 | 0 | 1 | 76 | 330 | 405 | 7.00E-23 | 111 |
| gi|19006458|gb|BM693200.1| | 93.42 | 76 | 5 | 0 | 1 | 76 | 282 | 207 | 7.00E-23 | 111 |
| gi|19721538|gb|BM996637.1| | 93.42 | 76 | 5 | 0 | 1 | 76 | 321 | 396 | 7.00E-23 | 111 |
| gi|23274374|gb|BU608159.1| | 93.42 | 76 | 5 | 0 | 1 | 76 | 326 | 401 | 7.00E-23 | 111 |
| gi|2784598|gb|AA743782.1| | 93.42 | 76 | 5 | 0 | 1 | 76 | 110 | 185 | 7.00E-23 | 111 |
| gi|2876039|gb|AA804638.1| | 93.42 | 76 | 5 | 0 | 1 | 76 | 318 | 393 | 7.00E-23 | 111 |
| gi|3933745|gb|AI290971.1| | 93.42 | 76 | 5 | 0 | 1 | 76 | 311 | 386 | 7.00E-23 | 111 |
| gi|4990875|gb|AI702975.1| | 93.42 | 76 | 5 | 0 | 1 | 76 | 307 | 382 | 7.00E-23 | 111 |
| gi|5454573|gb|AI832593.1| | 93.42 | 76 | 5 | 0 | 1 | 76 | 309 | 384 | 7.00E-23 | 111 |
| gi|7320253|gb|AW615067.1| | 93.42 | 76 | 5 | 0 | 1 | 76 | 313 | 388 | 7.00E-23 | 111 |
| gi|8167811|gb|AW976581.1| | 93.42 | 76 | 5 | 0 | 1 | 76 | 307 | 382 | 7.00E-23 | 111 |
| gi|8359944|gb|BE042891.1| | 93.42 | 76 | 5 | 0 | 1 | 76 | 307 | 382 | 7.00E-23 | 111 |
| gi|52721466|gb|CV371411.1| | 95.52 | 67 | 3 | 0 | 14 | 80 | 236 | 170 | 3.00E-22 | 109 |
| gi|32004424|emb|BX492684.1| | 92.41 | 79 | 6 | 0 | 2 | 80 | 382 | 304 | 3.00E-22 | 109 |
| gi|2907387|gb|AA833659.1| | 93.24 | 74 | 5 | 0 | 3 | 76 | 199 | 126 | 1.00E-21 | 107 |
| gi|52700258|gb|CV350203.1| | 93.24 | 74 | 5 | 0 | 2 | 75 | 551 | 624 | 1.00E-21 | 107 |
| gi|6837361|gb|AW340735.1| | 93.24 | 74 | 5 | 0 | 3 | 76 | 216 | 143 | 1.00E-21 | 107 |
| gi|7039615|gb|AW469509.1| | 93.24 | 74 | 5 | 0 | 3 | 76 | 216 | 143 | 1.00E-21 | 107 |
| gi|3400022|gb|AI073378.1| | 91.25 | 80 | 7 | 0 | 1 | 80 | 241 | 320 | 2.00E-20 | 103 |
| gi|46547768|gb|CN478769.1| | 91.25 | 80 | 7 | 0 | 1 | 80 | 255 | 334 | 2.00E-20 | 103 |
| gi|20494289|gb|BQ269223.1| | 92 | 75 | 6 | 0 | 1 | 75 | 483 | 409 | 6.00E-20 | 101 |
| gi|44842622|gb|CK825697.1| | 92 | 75 | 6 | 0 | 1 | 75 | 470 | 396 | 6.00E-20 | 101 |
| gi|45695156|emb|AL519606.3| | 94.12 | 68 | 3 | 1 | 13 | 80 | 747 | 681 | 3.00E-19 | 99.6 |
| gi|52811228|gb|CV415725.1| | 90.91 | 77 | 7 | 0 | 4 | 80 | 190 | 266 | 1.00E-18 | 97.6 |
| gi|2908283|gb|AA834684.1| | 94.12 | 68 | 3 | 1 | 5 | 72 | 137 | 203 | 4.00E-18 | 95.6 |
| gi|13292606|gb|BG399158.1| | 90.79 | 76 | 7 | 0 | 5 | 80 | 232 | 157 | 4.00E-18 | 95.6 |
| gi|52653216|gb|CV330002.1| | 90.79 | 76 | 7 | 0 | 5 | 80 | 163 | 88 | 4.00E-18 | 95.6 |
| gi|8061011|gb|AW896806.1| | 90.79 | 76 | 7 | 0 | 1 | 76 | 289 | 214 | 4.00E-18 | 95.6 |
| gi|52667308|gb|CV344094.1| | 92.11 | 76 | 5 | 1 | 1 | 76 | 280 | 206 | 4.00E-18 | 95.6 |
| gi|5438416|gb|AI819337.1| | 90 | 80 | 8 | 0 | 1 | 80 | 241 | 320 | 4.00E-18 | 95.6 |
| gi|7946376|gb|AW850859.1| | 90 | 80 | 8 | 0 | 1 | 80 | 170 | 249 | 4.00E-18 | 95.6 |
| gi|12766146|gb|BG256330.1| | 92.5 | 80 | 4 | 2 | 1 | 80 | 385 | 308 | 4.00E-18 | 95.6 |
| gi|27846682|emb|BX105680.1| | 91.55 | 71 | 6 | 0 | 8 | 78 | 364 | 434 | 2.00E-17 | 93.7 |
| gi|3837536|gb|AI242139.1| | 91.55 | 71 | 6 | 0 | 8 | 78 | 269 | 199 | 2.00E-17 | 93.7 |
| gi|58568449|dbj|BP395858.1| | 90.54 | 74 | 7 | 0 | 7 | 80 | 284 | 211 | 6.00E-17 | 91.7 |
| gi|6602709|emb|AL134522.1| | 93.24 | 74 | 3 | 2 | 4 | 77 | 28 | 99 | 6.00E-17 | 91.7 |
| gi|91749404|gb|EB386059.1| | 91.3 | 69 | 6 | 0 | 11 | 79 | 162 | 94 | 2.00E-16 | 89.7 |
| gi|32005544|emb|BX493226.1| | 92.75 | 69 | 4 | 1 | 7 | 75 | 216 | 149 | 2.00E-16 | 89.7 |
| gi|14321058|gb|BG926535.1| | 90.41 | 73 | 7 | 0 | 2 | 74 | 661 | 589 | 2.00E-16 | 89.7 |
| gi|52707894|gb|CV357839.1| | 90.41 | 73 | 7 | 0 | 2 | 74 | 127 | 55 | 2.00E-16 | 89.7 |
| gi|82333517|dbj|DA902558.1| | 90.91 | 77 | 6 | 1 | 1 | 77 | 193 | 118 | 2.00E-16 | 89.7 |
| gi|12120877|gb|BF772977.1| | 90.12 | 81 | 7 | 1 | 1 | 80 | 235 | 155 | 2.00E-16 | 89.7 |
| gi|12120883|gb|BF772983.1| | 90.12 | 81 | 7 | 1 | 1 | 80 | 236 | 156 | 2.00E-16 | 89.7 |
| gi|5110886|gb|AI742598.1| | 90.12 | 81 | 7 | 1 | 1 | 80 | 241 | 321 | 2.00E-16 | 89.7 |
| gi|1885842|gb|AA250882.1| | 92.19 | 64 | 5 | 0 | 1 | 64 | 41 | 104 | 1.00E-15 | 87.7 |
| gi|82341158|dbj|DB016887.1| | 92.19 | 64 | 5 | 0 | 1 | 64 | 188 | 251 | 1.00E-15 | 87.7 |
| gi|10200151|gb|BE778953.1| | 90.28 | 72 | 7 | 0 | 9 | 80 | 149 | 220 | 1.00E-15 | 87.7 |
| gi|13339103|gb|BG432597.1| | 89.47 | 76 | 8 | 0 | 5 | 80 | 516 | 441 | 1.00E-15 | 87.7 |
| gi|13343062|gb|BG436556.1| | 89.47 | 76 | 8 | 0 | 1 | 76 | 300 | 375 | 1.00E-15 | 87.7 |
| gi|15164200|emb|AL600694.1| | 89.47 | 76 | 8 | 0 | 1 | 76 | 324 | 399 | 1.00E-15 | 87.7 |
| gi|18983536|gb|BM673638.1| | 89.47 | 76 | 8 | 0 | 5 | 80 | 116 | 41 | 1.00E-15 | 87.7 |
| gi|19005651|gb|BM692393.1| | 89.47 | 76 | 8 | 0 | 5 | 80 | 185 | 260 | 1.00E-15 | 87.7 |
| gi|2834284|gb|AA774950.1| | 89.47 | 76 | 8 | 0 | 1 | 76 | 221 | 146 | 1.00E-15 | 87.7 |
| gi|11977833|gb|BF692425.1| | 90.79 | 76 | 6 | 1 | 1 | 76 | 386 | 460 | 1.00E-15 | 87.7 |
| gi|13452873|gb|BG491361.1| | 90.79 | 76 | 6 | 1 | 1 | 76 | 8 | 82 | 1.00E-15 | 87.7 |
| gi|13580923|gb|BG573270.1| | 90.79 | 76 | 6 | 1 | 1 | 76 | 290 | 364 | 1.00E-15 | 87.7 |
| gi|19727271|gb|BQ002371.1| | 90.79 | 76 | 6 | 1 | 1 | 76 | 400 | 326 | 1.00E-15 | 87.7 |
| gi|24776874|gb|CA414223.1| | 90.79 | 76 | 6 | 1 | 1 | 76 | 400 | 326 | 1.00E-15 | 87.7 |
| gi|27932373|gb|CB106566.1| | 90.79 | 76 | 6 | 1 | 1 | 76 | 13 | 87 | 1.00E-15 | 87.7 |
| gi|28365225|gb|CB243581.1| | 90.79 | 76 | 6 | 1 | 1 | 76 | 29 | 103 | 1.00E-15 | 87.7 |
| gi|43429246|emb|BX952415.1| | 90.79 | 76 | 6 | 1 | 5 | 80 | 89 | 163 | 1.00E-15 | 87.7 |
| gi|43425548|emb|BX951140.1| | 88.75 | 80 | 9 | 0 | 1 | 80 | 136 | 57 | 1.00E-15 | 87.7 |
| gi|3038959|gb|AA903836.1| | 90 | 80 | 7 | 1 | 1 | 80 | 74 | 152 | 1.00E-15 | 87.7 |
| gi|5543963|gb|AI869995.1| | 90 | 80 | 7 | 1 | 1 | 80 | 450 | 372 | 1.00E-15 | 87.7 |
| gi|7668921|gb|AW753989.1| | 91.04 | 67 | 6 | 0 | 12 | 78 | 421 | 487 | 4.00E-15 | 85.7 |
| gi|7668972|gb|AW754040.1| | 91.04 | 67 | 6 | 0 | 12 | 78 | 421 | 487 | 4.00E-15 | 85.7 |
| gi|8046501|gb|AW884489.1| | 88.61 | 79 | 9 | 0 | 2 | 80 | 132 | 210 | 4.00E-15 | 85.7 |
| gi|14466558|gb|BI059028.1| | 90 | 70 | 7 | 0 | 3 | 72 | 129 | 198 | 1.00E-14 | 83.8 |
| gi|81125345|dbj|DA460339.1| | 90 | 70 | 7 | 0 | 3 | 72 | 349 | 280 | 1.00E-14 | 83.8 |
| gi|27845181|emb|BX102210.1| | 89.74 | 78 | 7 | 1 | 1 | 77 | 407 | 330 | 1.00E-14 | 83.8 |
| gi|31915369|emb|BX479525.1| | 89.04 | 73 | 8 | 0 | 4 | 76 | 130 | 202 | 6.00E-14 | 81.8 |
| gi|66791763|dbj|BP425510.1| | 89.04 | 73 | 8 | 0 | 4 | 76 | 184 | 256 | 6.00E-14 | 81.8 |
| gi|685935|gb|T71414.1| | 88.16 | 76 | 9 | 0 | 1 | 76 | 11 | 86 | 6.00E-14 | 81.8 |
| gi|711241|gb|T82953.1| | 88.16 | 76 | 9 | 0 | 1 | 76 | 11 | 86 | 6.00E-14 | 81.8 |
| gi|1404173|gb|W88623.1| | 87.5 | 80 | 10 | 0 | 1 | 80 | 146 | 67 | 6.00E-14 | 81.8 |
| gi|1891141|gb|AA257012.1| | 88.89 | 81 | 8 | 1 | 1 | 80 | 248 | 168 | 6.00E-14 | 81.8 |
| gi|81181343|dbj|DA639796.1| | 90.62 | 64 | 6 | 0 | 1 | 64 | 16 | 79 | 2.00E-13 | 79.8 |
| gi|8058080|gb|AW893875.1| | 92.19 | 64 | 4 | 1 | 1 | 64 | 471 | 409 | 2.00E-13 | 79.8 |
| gi|14372680|gb|BG954509.1| | 90.28 | 72 | 6 | 1 | 9 | 80 | 238 | 168 | 2.00E-13 | 79.8 |
| gi|79163886|dbj|DA105807.1| | 90.28 | 72 | 6 | 1 | 9 | 80 | 387 | 317 | 2.00E-13 | 79.8 |
| gi|8623066|gb|BE160345.1| | 90.28 | 72 | 6 | 1 | 9 | 80 | 94 | 164 | 2.00E-13 | 79.8 |
| gi|8623148|gb|BE160427.1| | 90.28 | 72 | 6 | 1 | 9 | 80 | 94 | 164 | 2.00E-13 | 79.8 |
| gi|2162267|gb|AA448597.1| | 88.16 | 76 | 9 | 0 | 1 | 76 | 343 | 418 | 2.00E-13 | 79.8 |
| gi|80799866|dbj|DA505931.1| | 88.16 | 76 | 9 | 0 | 1 | 76 | 121 | 196 | 2.00E-13 | 79.8 |
| gi|21855046|gb|BQ716149.1| | 89.47 | 76 | 7 | 1 | 1 | 76 | 119 | 193 | 2.00E-13 | 79.8 |
| gi|24805094|gb|CA440674.1| | 89.47 | 76 | 7 | 1 | 1 | 76 | 400 | 326 | 2.00E-13 | 79.8 |
| gi|83480277|dbj|DB358036.1| | 89.47 | 76 | 7 | 1 | 1 | 76 | 382 | 308 | 2.00E-13 | 79.8 |
| gi|3056341|gb|AA916949.1| | 87.5 | 80 | 10 | 0 | 1 | 80 | 230 | 309 | 2.00E-13 | 79.8 |
| gi|1764951|gb|AA181484.1| | 88.75 | 80 | 8 | 1 | 2 | 80 | 360 | 281 | 2.00E-13 | 79.8 |
| gi|8054117|gb|AW889912.1| | 88.75 | 80 | 8 | 1 | 1 | 80 | 149 | 227 | 2.00E-13 | 79.8 |
| gi|83532058|dbj|DB333866.1| | 88.75 | 80 | 8 | 1 | 1 | 80 | 65 | 143 | 2.00E-13 | 79.8 |
| gi|10107714|gb|BE719449.1| | 88.73 | 71 | 8 | 0 | 1 | 71 | 623 | 553 | 9.00E-13 | 77.8 |
| gi|504666|dbj|D20846.1| | 90.14 | 71 | 6 | 1 | 1 | 70 | 191 | 121 | 9.00E-13 | 77.8 |
| gi|14393270|gb|BG989200.1| | 89.33 | 75 | 7 | 1 | 6 | 80 | 304 | 231 | 9.00E-13 | 77.8 |
| gi|31446439|gb|CD514721.1| | 89.33 | 75 | 7 | 1 | 6 | 80 | 13 | 86 | 9.00E-13 | 77.8 |
| gi|2617003|gb|AA663012.1| | 88.46 | 78 | 7 | 1 | 5 | 80 | 105 | 28 | 9.00E-13 | 77.8 |
| gi|90847359|dbj|DB577513.1| | 87.65 | 81 | 7 | 1 | 1 | 78 | 52 | 132 | 9.00E-13 | 77.8 |
| gi|83241952|dbj|DB315742.1| | 90 | 70 | 6 | 1 | 11 | 79 | 73 | 142 | 4.00E-12 | 75.8 |
| gi|91749668|gb|EB386323.1| | 90 | 70 | 6 | 1 | 9 | 77 | 226 | 157 | 4.00E-12 | 75.8 |
| gi|10918992|dbj|AV761144.1| | 88.46 | 78 | 8 | 1 | 3 | 79 | 236 | 313 | 4.00E-12 | 75.8 |
| gi|78737823|dbj|DA326471.1| | 88.46 | 78 | 8 | 1 | 3 | 80 | 82 | 6 | 4.00E-12 | 75.8 |
| gi|83199537|dbj|DB235269.1| | 85.88 | 85 | 6 | 1 | 2 | 80 | 480 | 396 | 4.00E-12 | 75.8 |
| gi|7668920|gb|AW753988.1| | 89.23 | 65 | 7 | 0 | 14 | 78 | 91 | 27 | 1.00E-11 | 73.8 |
| gi|7668971|gb|AW754039.1| | 89.23 | 65 | 7 | 0 | 14 | 78 | 91 | 27 | 1.00E-11 | 73.8 |
| gi|83486421|dbj|DB358889.1| | 89.23 | 65 | 7 | 0 | 16 | 80 | 329 | 393 | 1.00E-11 | 73.8 |
| gi|2328991|gb|AA558514.1| | 87.67 | 73 | 9 | 0 | 4 | 76 | 115 | 43 | 1.00E-11 | 73.8 |
| gi|81156387|dbj|DA383600.1| | 87.67 | 73 | 9 | 0 | 4 | 76 | 516 | 588 | 1.00E-11 | 73.8 |
| gi|8165082|gb|AW973998.1| | 87.67 | 73 | 9 | 0 | 4 | 76 | 244 | 172 | 1.00E-11 | 73.8 |
| gi|23373989|gb|BU661807.1| | 88.16 | 76 | 7 | 1 | 5 | 80 | 85 | 158 | 1.00E-11 | 73.8 |
| gi|3896467|gb|AI274199.1| | 88.16 | 76 | 7 | 1 | 1 | 74 | 74 | 149 | 1.00E-11 | 73.8 |
| gi|5054918|gb|AI733805.1| | 88.16 | 76 | 7 | 1 | 1 | 74 | 72 | 147 | 1.00E-11 | 73.8 |
| gi|82136433|dbj|DB047679.1| | 88.31 | 77 | 8 | 1 | 1 | 76 | 152 | 228 | 1.00E-11 | 73.8 |
| gi|33252132|gb|CF136688.1| | 90.62 | 64 | 5 | 1 | 1 | 64 | 30 | 92 | 6.00E-11 | 71.9 |
| gi|46922787|emb|BX405577.2| | 87.5 | 80 | 9 | 1 | 1 | 80 | 146 | 224 | 6.00E-11 | 71.9 |
| gi|46233530|emb|AL566894.3| | 86.3 | 73 | 9 | 1 | 1 | 73 | 526 | 455 | 2.00E-10 | 69.9 |
| gi|3872647|gb|AI264444.1| | 86.84 | 76 | 8 | 1 | 1 | 74 | 72 | 147 | 2.00E-10 | 69.9 |
| gi|33258518|gb|CF143074.1| | 86.25 | 80 | 7 | 1 | 1 | 80 | 184 | 259 | 2.00E-10 | 69.9 |
| gi|7111293|gb|AW499536.1| | 86.25 | 80 | 7 | 1 | 1 | 80 | 184 | 259 | 2.00E-10 | 69.9 |
| gi|7111295|gb|AW499537.1| | 86.25 | 80 | 7 | 1 | 1 | 80 | 184 | 259 | 2.00E-10 | 69.9 |
| gi|7111327|gb|AW499553.1| | 86.25 | 80 | 7 | 1 | 1 | 80 | 184 | 259 | 2.00E-10 | 69.9 |
| gi|7116331|gb|AW502136.1| | 86.25 | 80 | 7 | 1 | 1 | 80 | 184 | 259 | 2.00E-10 | 69.9 |
| gi|7116335|gb|AW502138.1| | 86.25 | 80 | 7 | 1 | 1 | 80 | 184 | 259 | 2.00E-10 | 69.9 |
| gi|3214298|gb|AI004788.1| | 85.71 | 84 | 8 | 1 | 1 | 80 | 128 | 45 | 2.00E-10 | 69.9 |
| gi|90648194|dbj|BY797461.2| | 85.71 | 84 | 8 | 1 | 1 | 80 | 347 | 430 | 2.00E-10 | 69.9 |
| gi|1486755|gb|AA022674.1| | 84.88 | 86 | 7 | 1 | 1 | 80 | 283 | 198 | 2.00E-10 | 69.9 |
| gi|1486863|gb|AA022709.1| | 84.88 | 86 | 7 | 1 | 1 | 80 | 101 | 186 | 2.00E-10 | 69.9 |
| gi|83124689|dbj|DB343577.1| | 86.49 | 74 | 10 | 0 | 4 | 77 | 405 | 478 | 9.00E-10 | 67.9 |
| gi|90938918|dbj|DB507251.1| | 87.84 | 74 | 8 | 1 | 1 | 74 | 74 | 2 | 9.00E-10 | 67.9 |
| gi|694186|gb|T76983.1| | 86.84 | 76 | 7 | 1 | 5 | 80 | 204 | 132 | 9.00E-10 | 67.9 |
| gi|83078449|dbj|DB106337.1| | 85.9 | 78 | 7 | 1 | 1 | 78 | 458 | 385 | 9.00E-10 | 67.9 |
| gi|83237080|dbj|DB354909.1| | 86.25 | 80 | 8 | 1 | 1 | 80 | 218 | 294 | 9.00E-10 | 67.9 |
| gi|12189868|gb|BF837652.1| | 88.41 | 69 | 7 | 1 | 1 | 69 | 148 | 215 | 4.00E-09 | 65.9 |
| gi|8167508|gb|AW976282.1| | 87.67 | 73 | 8 | 1 | 5 | 76 | 495 | 423 | 4.00E-09 | 65.9 |
| gi|14399447|gb|BG995377.1| | 85.71 | 77 | 11 | 0 | 4 | 80 | 227 | 151 | 4.00E-09 | 65.9 |
| gi|81108769|dbj|DA381665.1| | 85.9 | 78 | 7 | 1 | 1 | 78 | 487 | 414 | 4.00E-09 | 65.9 |
| gi|83190413|dbj|DB352537.1| | 87.65 | 81 | 8 | 2 | 1 | 80 | 312 | 391 | 4.00E-09 | 65.9 |
| gi|83517488|dbj|DB143470.1| | 86.84 | 76 | 9 | 1 | 1 | 76 | 116 | 42 | 1.00E-08 | 63.9 |
| gi|82338292|dbj|DB049879.1| | 85 | 80 | 12 | 0 | 1 | 80 | 166 | 87 | 1.00E-08 | 63.9 |
| gi|80933029|dbj|DA523524.1| | 86.25 | 80 | 10 | 1 | 1 | 80 | 468 | 390 | 1.00E-08 | 63.9 |

^1^BLASTN was used to search the human EST database with a full length Made1 element query sequence. Only hits that were ≥80% identical over ≥80% of the length of the element are reported. Hit identifiers (Genbank identification numbers and accessions) are shown followed by the BLAST statistics for each query-hit pair.
