# Supplementary material for: A Family of Human MicroRNA Genes from Miniature Inverted-Repeat Transposable Elements
Source: PLoS One. 2007 Feb 14;2(2):e203. doi: 10.1371/journal.pone.0000203 (PMC1784062; doi:10.1371/journal.pone.0000203)
Supplement: Table S2 — Over-represented GO biological process categories among genes with Made1 derived hsa-mir-548 target sites. (0.04 MB DOC) [file pone.0000203.s005.doc]

Table S2. **Over-represented GO biological process categories among genes with Made1 derived hsa-mir-548 target sites.**

| **GO ID^1^** | **Description^2^** | **Gene acc^3^** | **Obs^4^** | **Exp^5^** | ***P*-value^6^** |
| --- | --- | --- | --- | --- | --- |
| GO:0000087 | M phase of mitotic cell cycle | ENSG00000130177  ENSG00000086827*  ENSG00000004897* | 3 | 0.44 | 9.42E-03 |
| GO:0007067 | mitosis | ENSG00000130177  ENSG00000086827*  ENSG00000004897* | 3 | 0.43 | 9.06E-03 |
| GO:0007088 | regulation of mitosis | ENSG00000130177  ENSG00000086827* | 2 | 0.12 | 6.39E-03 |
| GO:0006917 | induction of apoptosis | ENSG00000163161  ENSG00000171132*  ENSG00000004468 | 3 | 0.44 | 9.42E-03 |
| GO:0012502 | induction of programmed cell death | ENSG00000163161  ENSG00000171132*  ENSG00000004468 | 3 | 0.44 | 9.42E-03 |
| GO:0008283 | cell proliferation | ENSG00000076716  ENSG00000112038*  ENSG00000143125  ENSG00000125657  ENSG00000130177  ENSG00000004897* | 6 | 1.58 | 4.47E-03 |
| GO:0007059 | chromosome segregation | ENSG00000163535  ENSG00000086827* | 2 | 0.11 | 5.17E-03 |

^1^ GO biological process category ID

^2^ Functional description for the GO category

^3^ The list of Ensembl gene accessions in the GO category, * indicates genes that are down-regulated in colorectal cancer tissue

^4^ Observed gene number in the GO category

^5^ Expected gene number in the GO category

^6^ *P*-value showing significance of enrichment for the GO category based on the hypergeometric test
