# Supplementary material for: A Family of Human MicroRNA Genes from Miniature Inverted-Repeat Transposable Elements
Source: PLoS One. 2007 Feb 14;2(2):e203. doi: 10.1371/journal.pone.0000203 (PMC1784062; doi:10.1371/journal.pone.0000203)
Supplement: Table S3 — Over-represented GO biological process categories among genes with miRanda predicted hsa-mir-548 target sites that map to colorectal cancer down-regulated co-expression clusters (i.e. 12, 15 & 20 in Figure 6). (0.09 MB DOC) [file pone.0000203.s006.doc]

Table S3. **Over-represented GO biological process categories among genes with miRanda predicted hsa-mir-548 target sites that map to colorectal cancer down-regulated co-expression clusters (*i.e.* 12, 15 & 20 in Figure 6).**

| **GO ID^1^** | **Description^2^** | **Gene acc^3^** | **Obs^4^** | **Exp^5^** | ***P*-value^6^** |
| --- | --- | --- | --- | --- | --- |
| GO:0007155 | cell adhesion | ENSG00000179776  ENSG00000040731  ENSG00000154162  ENSG00000133800  ENSG00000073712  ENSG00000138080  ENSG00000018236  ENSG00000038427  ENSG00000170989  ENSG00000146648  ENSG00000128536  ENSG00000087303  ENSG00000115414  ENSG00000102290  ENSG00000164171  ENSG00000158887  ENSG00000067141  ENSG00000124215  ENSG00000107562  ENSG00000112378  ENSG00000143341  ENSG00000164199  ENSG00000077522  ENSG00000104415  ENSG00000163347  ENSG00000154655  ENSG00000198542 | 27 | 10.05 | 2.61E-06 |
| GO:0016337 | cell-cell adhesion | ENSG00000179776  ENSG00000040731  ENSG00000154162  ENSG00000146648  ENSG00000128536  ENSG00000102290  ENSG00000158887  ENSG00000124215  ENSG00000164199  ENSG00000163347 | 10 | 3.45 | 2.48E-03 |
| GO:0007156 | homophilic cell adhesion | ENSG00000179776  ENSG00000040731  ENSG00000154162  ENSG00000128536  ENSG00000102290  ENSG00000158887  ENSG00000124215 | 7 | 2.16 | 5.99E-03 |
| GO:0031589 | cell-substrate adhesion | ENSG00000133800  ENSG00000087303  ENSG00000164171  ENSG00000077522  ENSG00000198542 | 5 | 0.9 | 2.03E-03 |
| GO:0007160 | cell-matrix adhesion | ENSG00000133800  ENSG00000087303  ENSG00000164171  ENSG00000077522  ENSG00000198542 | 5 | 0.9 | 2.03E-03 |
| GO:0007154 | cell communication | ENSG00000064989  ENSG00000153208  ENSG00000145632  ENSG00000166073  ENSG00000184984  ENSG00000080644  ENSG00000147432  ENSG00000135902  ENSG00000108018  ENSG00000018236  ENSG00000174429  ENSG00000169676  ENSG00000170989  ENSG00000146648  ENSG00000140009  ENSG00000151348  ENSG00000138685  ENSG00000115641  ENSG00000115414  ENSG00000113327  ENSG00000091844  ENSG00000164949  ENSG00000146072  ENSG00000135821  ENSG00000127920  ENSG00000177464  ENSG00000132975  ENSG00000064652  ENSG00000171189  ENSG00000095752  ENSG00000164171  ENSG00000183111  ENSG00000182634  ENSG00000113594  ENSG00000101665  ENSG00000116141  ENSG00000124089  ENSG00000143198  ENSG00000158887  ENSG00000067141  ENSG00000134259  ENSG00000170485  ENSG00000133636  ENSG00000165588  ENSG00000169860  ENSG00000167941  ENSG00000115252  ENSG00000154678  ENSG00000172572  ENSG00000113448  ENSG00000108551  ENSG00000156475  ENSG00000156218  ENSG00000144724  ENSG00000115665  ENSG00000166592  ENSG00000107562  ENSG00000196632  ENSG00000196781  ENSG00000105989  ENSG00000175868  ENSG00000152284  ENSG00000182880  ENSG00000164199  ENSG00000104415  ENSG00000049246  ENSG00000124104  ENSG00000078043  ENSG00000165970  ENSG00000149305  ENSG00000170579  ENSG00000198542  ENSG00000137962  ENSG00000198752  ENSG00000064692  ENSG00000198929 | 76 | 51.71 | 1.01E-04 |
| GO:0007267 | cell-cell signaling | ENSG00000153208  ENSG00000166073  ENSG00000147432  ENSG00000169676  ENSG00000140009  ENSG00000138685  ENSG00000135821  ENSG00000171189  ENSG00000095752  ENSG00000158887  ENSG00000067141  ENSG00000134259  ENSG00000115665  ENSG00000107562  ENSG00000104415  ENSG00000165970  ENSG00000149305  ENSG00000170579  ENSG00000064692  ENSG00000198929 | 20 | 8.45 | 3.13E-04 |
| GO:0019226 | transmission of nerve impulse | ENSG00000166073  ENSG00000147432  ENSG00000169676  ENSG00000135821  ENSG00000171189  ENSG00000158887  ENSG00000115665  ENSG00000165970  ENSG00000149305  ENSG00000170579  ENSG00000064692  ENSG00000198929 | 12 | 3.79 | 4.30E-04 |
| GO:0007268 | synaptic transmission | ENSG00000166073  ENSG00000147432  ENSG00000169676  ENSG00000135821  ENSG00000171189  ENSG00000158887  ENSG00000115665  ENSG00000165970  ENSG00000149305  ENSG00000170579  ENSG00000064692  ENSG00000198929 | 12 | 3.64 | 3.02E-04 |
| GO:0001505 | regulation of neurotransmitter levels | ENSG00000135821  ENSG00000115665  ENSG00000064692  ENSG00000198929 | 4 | 0.73 | 5.84E-03 |
| GO:0007165 | signal transduction | ENSG00000064989  ENSG00000153208  ENSG00000145632  ENSG00000166073  ENSG00000184984  ENSG00000080644  ENSG00000147432  ENSG00000135902  ENSG00000108018  ENSG00000018236  ENSG00000174429  ENSG00000169676  ENSG00000170989  ENSG00000146648  ENSG00000140009  ENSG00000151348  ENSG00000138685  ENSG00000115641  ENSG00000115414  ENSG00000113327  ENSG00000091844  ENSG00000164949  ENSG00000146072  ENSG00000127920  ENSG00000177464  ENSG00000132975  ENSG00000064652  ENSG00000171189  ENSG00000164171  ENSG00000183111  ENSG00000182634  ENSG00000113594  ENSG00000101665  ENSG00000116141  ENSG00000124089  ENSG00000143198  ENSG00000170485  ENSG00000133636  ENSG00000165588  ENSG00000169860  ENSG00000167941  ENSG00000115252  ENSG00000154678  ENSG00000172572  ENSG00000113448  ENSG00000108551  ENSG00000156475  ENSG00000156218  ENSG00000144724  ENSG00000166592  ENSG00000107562  ENSG00000196632  ENSG00000196781  ENSG00000105989  ENSG00000175868  ENSG00000152284  ENSG00000182880  ENSG00000164199  ENSG00000104415  ENSG00000049246  ENSG00000124104  ENSG00000078043  ENSG00000198542  ENSG00000137962  ENSG00000198752 | 65 | 47.47 | 2.90E-03 |
| GO:0051056 | regulation of small GTPase mediated signal transduction | ENSG00000174429  ENSG00000183111  ENSG00000198752 | 3 | 0.27 | 2.38E-03 |
| GO:0035023 | regulation of Rho protein signal transduction | ENSG00000174429  ENSG00000183111 | 2 | 0.06 | 1.52E-03 |
| GO:0007266 | Rho protein signal transduction | ENSG00000174429  ENSG00000183111  ENSG00000137962 | 3 | 0.39 | 6.51E-03 |
| GO:0009966 | regulation of signal transduction | ENSG00000145632  ENSG00000174429  ENSG00000091844  ENSG00000183111  ENSG00000165588  ENSG00000167941  ENSG00000196781  ENSG00000152284  ENSG00000198752 | 9 | 3.31 | 6.04E-03 |
| GO:0006575 | amino acid derivative metabolism | ENSG00000129596  ENSG00000131480  ENSG00000115665  ENSG00000064692 | 4 | 0.82 | 9.08E-03 |
| GO:0009250 | glucan biosynthesis | ENSG00000111713  ENSG00000056998 | 2 | 0.15 | 8.65E-03 |
| GO:0005978 | glycogen biosynthesis | ENSG00000111713  ENSG00000056998 | 2 | 0.15 | 8.65E-03 |
| GO:0007417 | central nervous system development | ENSG00000061676  ENSG00000171189  ENSG00000170485  ENSG00000165588  ENSG00000134595  ENSG00000043355 | 6 | 1.82 | 9.91E-03 |
| GO:0007596 | blood coagulation | ENSG00000095752  ENSG00000164171  ENSG00000169860  ENSG00000143341  ENSG00000154655 | 5 | 1.29 | 9.39E-03 |
| GO:0051260 | protein homooligomerization | ENSG00000187134  ENSG00000077522 | 2 | 0.15 | 8.65E-03 |
| GO:0050952 | sensory perception of electrical stimulus | ENSG00000182634  ENSG00000182880 | 2 | 0 | 0.00E+00 |
| GO:0050978 | magnetoreception, using electrical stimulus | ENSG00000182634  ENSG00000182880 | 2 | 0 | 0.00E+00 |
| GO:0050954 | sensory perception of mechanical stimulus | ENSG00000153208  ENSG00000115380  ENSG00000131480  ENSG00000140522  ENSG00000143341  ENSG00000164199 | 6 | 1.58 | 5.03E-03 |
| GO:0050979 | magnetoreception, using mechanical stimulus | ENSG00000153208  ENSG00000115380  ENSG00000131480  ENSG00000140522  ENSG00000143341  ENSG00000164199 | 6 | 0 | 0.00E+00 |
| GO:0019233 | sensory perception of pain | ENSG00000165091  ENSG00000164199  ENSG00000136156 | 3 | 0.03 | 1.41E-06 |
| GO:0050966 | detection of mechanical stimulus during sensory perception of pain | ENSG00000165091  ENSG00000164199  ENSG00000136156 | 3 | 0 | 0.00E+00 |
| GO:0051341 | regulation of oxidoreductase activity | ENSG00000146648  ENSG00000198929 | 2 | 0.06 | 1.52E-03 |
| GO:0050999 | regulation of nitric-oxide synthase activity | ENSG00000146648  ENSG00000198929 | 2 | 0.06 | 1.52E-03 |

^1^ GO biological process category ID

^2^ Functional description for the GO category

^3^ The list of Ensembl gene accessions in the GO category

^4^ Observed gene number in the GO category

^5^ Expected gene number in the GO category

^6^ *P*-value showing significance of enrichment for the GO category based on the hypergeometric test
