# Supplementary material for: A Family of Human MicroRNA Genes from Miniature Inverted-Repeat Transposable Elements
Source: PLoS One. 2007 Feb 14;2(2):e203. doi: 10.1371/journal.pone.0000203 (PMC1784062; doi:10.1371/journal.pone.0000203)
Supplement: Table S4 — Putative hsa-mir-548 target genes previously implicated as being involved in colorectal cancer by microarray expression profiling. (0.20 MB DOC) [file pone.0000203.s007.doc]

Table S4. **Putative hsa-mir-548 target genes previously implicated as being involved in colorectal cancer by microarray expression profiling.**

| **Accn^1^** | **Ref^2^** | **Name^3^** | **Status^4^** | **Target^5^** | ***P*-value^6^** |
| --- | --- | --- | --- | --- | --- |
| ENST00000282050 | [1] | ATP synthase alpha chain, mitochondrial precursor (EC 3.6.3.14) | down | a | 1.73E-05 |
| ENST00000219660 | [1] | Aquaporin-8 (AQP-8) | down | b | 0.0050 |
| ENST00000262825 | [2] | Cytokine receptor common beta chain precursor (GM-CSF/IL-3/IL-5 receptor common beta-chain) (CD131 antigen) (CDw131) | down | b | 0.0006 |
| ENST00000201031 | [2] | Transcription factor AP-2 gamma (AP2-gamma) (Activating enhancer- binding protein 2 gamma) (Transcription factor ERF-1) | down | a,b,c,d | 0.0006 |
| ENST00000241261 | [2] | Tumor necrosis factor ligand superfamily member 10 (TNF-related apoptosis-inducing ligand) (TRAIL protein) (Apo-2 ligand) (Apo-2L) (CD253 antigen) | down | a | 0.0315 |
| ENST00000360121 | [2] | Leukosialin precursor (Leucocyte sialoglycoprotein) (Sialophorin) (Galactoglycoprotein) (GALGP) (CD43 antigen) | down | a,c | 0.0018 |
| ENST00000360876 | [2] | Eukaryotic translation initiation factor 3 subunit 9 (eIF-3 eta) (eIF3 p116) (eIF3 p110) (eIF3b) (Prt1 homolog) (hPrt1) | up | a | 0.0176 |
| ENST00000368083 | [3] | Arginase-1 (EC 3.5.3.1) (Type I arginase) (Liver-type arginase) | down | c | 0.0308 |
| ENST00000344548 | [3] | Cell division control protein 42 homolog precursor (G25K GTP-binding protein) | down | c | 0.0455 |
| ENST00000379328 | [3] | Trans-acting T-cell-specific transcription factor GATA-3 (GATA-binding factor 3) | down | a | 0.0012 |
| ENST00000285900 | [3] | Glutamate receptor 1 precursor (GluR-1) (GluR-A) (GluR-K1) (Glutamate receptor ionotropic, AMPA 1) (AMPA-selective glutamate receptor 1) | down | d | 0.0058 |
| ENST00000328245 | [3] | Heat shock factor protein 1 (HSF 1) (Heat shock transcription factor 1) (HSTF 1) | down | c | 1.03E-05 |
| ENST00000227752 | [3] | Interleukin-10 receptor alpha chain precursor (IL-10R-A) (IL-10R1) (CDw210a antigen) | down | d | 0.0206 |
| ENST00000371794 | [3] | Noelin precursor (Neuronal olfactomedin-related ER localized protein) (Olfactomedin-1) | down | d | 0.0410 |
| ENST00000334661 | [3] | 1-phosphatidylinositol-4,5-bisphosphate phosphodiesterase delta 1 (EC 3.1.4.11) (Phosphoinositide phospholipase C) (PLC-delta-1) (Phospholipase C-delta-1) (PLC-III) | down | c | 0.0198 |
| ENST00000229390 | [3] | Splicing factor, arginine/serine-rich 9 (Pre-mRNA-splicing factor SRp30C) | down | a | 0.0005 |
| ENST00000340600 | [3] | Suppressor of cytokine signaling 2 (SOCS-2) (Cytokine-inducible SH2 protein 2) (CIS-2) (STAT-induced STAT inhibitor 2) (SSI-2) | down | b | 0.0056 |
| ENST00000288207 | [3] | G2/mitotic-specific cyclin-B2 | up | a,b,c,d | 0.0010 |
| ENST00000264161 | [3] | Aspartyl-tRNA synthetase (EC 6.1.1.12) (Aspartate--tRNA ligase) (AspRS) | up | c | 0.0121 |
| ENST00000309268 | [3] | Elongation factor 1-alpha 1 (EF-1-alpha-1) (Elongation factor 1 A-1) (eEF1A-1) (Elongation factor Tu) (EF-Tu) | up | a | 0.0252 |
| ENST00000319974 | [3] | no description (ets variant gene 4 (E1A enhancer binding protein, E1AF)) | up | a | 0.0025 |
| ENST00000302068 | [3] | Fibrinogen beta chain precursor [Contains: Fibrinopeptide B] | up | a,c | 0.0053 |
| ENST00000341048 | [3] | no description (interleukin 6 signal transducer (gp130, oncostatin M receptor)) | up | a | 0.0004 |
| ENST00000296585 | [3] | Integrin alpha-2 precursor (Platelet membrane glycoprotein Ia) (GPIa) (Collagen receptor) (VLA-2 alpha chain) (CD49b antigen) | up | d | 0.0088 |
| ENST00000260302 | [3] | Collagenase 3 precursor (EC 3.4.24.-) (Matrix metalloproteinase-13) (MMP-13) | up | b | 0.0197 |
| ENST00000296930 | [3] | Nucleophosmin (NPM) (Nucleolar phosphoprotein B23) (Numatrin) (Nucleolar protein NO38) | up | a | 0.0015 |
| ENST00000216392 | [3] | Glycogen phosphorylase, liver form (EC 2.4.1.1) | up | b,c,d | 0.0467 |
| ENST00000370321 | [3] | 60S ribosomal protein L5 | up | c | 0.0032 |
| ENST00000265361 | [3] | Semaphorin-3C precursor (Semaphorin E) (Sema E) | up | d | 8.05E-05 |
| ENST00000244520 | [3] | U1 small nuclear ribonucleoprotein C (U1 snRNP protein C) (U1C protein) (U1-C) | up | a | 0.0001 |
| ENST00000273258 | [4] | PRA1 family protein 3 (ARL-6-interacting protein 5) (ADP-ribosylation- like factor 6-interacting protein 5) (Aip-5) (Glutamate transporter EAAC1-interacting protein) (GTRAP3-18) (Prenylated Rab acceptor protein 2) (Protein JWa) (Dermal papilla-derived pro | down | b | 0.0014 |
| ENST00000323456 | [4] | myotubularin related protein 4 | down | b | 0.0011 |
| ENST00000258428 | [4] | DNA repair protein REV1 (EC 2.7.7.-) (Rev1-like terminal deoxycytidyl transferase) (Alpha integrin-binding protein 80) (AIBP80) | down | c | 0.0011 |
| ENST00000326361 | [4] | Zinc finger protein 639 (Zinc finger protein ZASC1) (Zinc finger protein ANC_2H01) | up | a | 0.0015 |
| ENST00000259075 | [4] | TRAF family member-associated NF-kappa-B activator (TRAF-interacting protein) (I-TRAF) | up | b,c,d | 7.5E-05 |
| ENST00000262462 | [4] | Long-chain fatty acid transport protein 6 (Fatty acid transport protein 6) (FATP-6) (Very long-chain acyl-CoA synthetase homolog 1) (VLCSH1) (hVLCS-H1) (Fatty-acid-coenzyme A ligase, very long-chain 2) (Solute carrier family 27 member 6) | up | a,c | 6.4E-05 |
| ENST00000307633 | [4] | Histidyl-tRNA synthetase (EC 6.1.1.21) (Histidine--tRNA ligase) (HisRS) | up | c | 0.0001 |
| ENST00000327304 | [4] | Exosome complex exonuclease RRP40 (EC 3.1.13.-) (Ribosomal RNA- processing protein 40) (Exosome component 3) (p10) | up | b,c,d | 0.0001 |
| ENST00000370986 | [4] | Growth arrest and DNA-damage-inducible protein GADD45 alpha (DNA- damage-inducible transcript 1) (DDIT1) | up | a | 0.0013 |
| ENST00000160827 | [4] | Kinesin-like protein KIF22 (Kinesin-like DNA-binding protein) (Kinesin-like protein 4) | up | a | 0.0031 |
| ENST00000230588 | [5] | Meprin A subunit alpha precursor (EC 3.4.24.18) (Endopeptidase-2) (N- benzoyl-L-tyrosyl-P-amino-benzoic acid hydrolase subunit alpha) (PABA peptide hydrolase) (PPH alpha) | down | a | 0.0012 |
| ENST00000162749 | [5] | Tumor necrosis factor receptor superfamily member 1A precursor (p60) (TNF-R1) (TNF-RI) (TNFR-I) (p55) (CD120a antigen) [Contains: Tumor necrosis factor receptor superfamily member 1A, membrane form; Tumor necrosis factor-binding protein 1 (TBPI)] | down | b | 0.0023 |
| ENST00000314355 | [5] | Cyclin-dependent kinases regulatory subunit 2 (CKS-2) | up | a | 0.0136 |
| ENST00000283646 | [6] | Ribose-5-phosphate isomerase (EC 5.3.1.6) (Phosphoriboisomerase) | down | a | 4.2E-05 |
| ENST00000356245 | [6] | Ras-GTPase-activating protein-binding protein 1 (EC 3.6.1.-) (ATP- dependent DNA helicase VIII) (GAP SH3-domain-binding protein 1) (G3BP- 1) (HDH-VIII) | up | b | 4.15E-05 |

^1^Ensembl transcript accession for putative hsa-mir-548 target genes

^2^Publication where the genes involvement in colorectal cancer was originally reported

^3^Name and brief description of the gene

^4^Expression status of the gene (up- or down-regulated) in colorectal cancer relative to normal tissue

^5^Paralog-specific hsa-mir-548 target site

^6^*P*-value associated with the hsa-mir-548 target sites

1. Takemasa I, Higuchi H, Yamamoto H, Sekimoto M, Tomita N, et al. (2001) Construction of preferential cDNA microarray specialized for human colorectal carcinoma: molecular sketch of colorectal cancer. Biochem Biophys Res Commun 285: 1244-1249.

2. Kitahara O, Furukawa Y, Tanaka T, Kihara C, Ono K, et al. (2001) Alterations of gene expression during colorectal carcinogenesis revealed by cDNA microarrays after laser-capture microdissection of tumor tissues and normal epithelia. Cancer Res 61: 3544-3549.

3. Bertucci F, Salas S, Eysteries S, Nasser V, Finetti P, et al. (2004) Gene expression profiling of colon cancer by DNA microarrays and correlation with histoclinical parameters. Oncogene 23: 1377-1391.

4. Kwon HC, Kim SH, Roh MS, Kim JS, Lee HS, et al. (2004) Gene expression profiling in lymph node-positive and lymph node-negative colorectal cancer. Dis Colon Rectum 47: 141-152.

5. Notterman DA, Alon U, Sierk AJ, Levine AJ (2001) Transcriptional gene expression profiles of colorectal adenoma, adenocarcinoma, and normal tissue examined by oligonucleotide arrays. Cancer Res 61: 3124-3130.

6. Shih W, Chetty R, Tsao MS (2005) Expression profiling by microarrays in colorectal cancer (Review). Oncol Rep 13: 517-524.
